# Do two-wheeler riders propel towards autonomous driving? A path-based probabilistic study in Indian context

**DOI:** 10.1016/j.heliyon.2024.e35664

**Published:** 2024-08-05

**Authors:** Suprabeet Datta, Gone Sankeerthana, B. Raghuram Kadali

**Affiliations:** a Transportation Division, National Institute of Technology Warangal, Hanamkonda, Telangana, 506004, India; b National Institute of Technology Warangal, Hanamkonda, Telangana, 506004, India; c Transportation Division, Department of Civil Engineering, National Institute of Technology Warangal, Hanamkonda, Telangana, 506004, India

**Keywords:** Two-wheeler riders, Autonomous vehicles, Intention to use, Perceived risks, Indian mixed traffic

## Abstract

Regardless of the rational benefits of autonomous vehicle adoption towards mitigating mobility goals in urban and sub-urban areas of middle-income countries, adoption of such technology remains thought-provoking. Two wheeled vehicles are often utilized extensively all over the world for short distance trips in sub-urban areas. The present study investigates perceived risks of using four wheeled autonomous vehicles by captive two-wheeler riders for long and short distance trips in densely populated Indian cities. Theoretical autonomous vehicle technology acceptance models were modified considering Indian perceptions in this study. Offline stated preference responses were collected from various universities, open public spaces and governmental organizations of various cities within India. Structural equation models (SEM) and Random Forest (RF) were employed to generate meaningful insights from the surveys conducted. Random Forest (RF) analysis concluded that bicycle riders show medium to low levels of trust towards behavioural adoption intention of autonomous vehicles. According to proposed structural equation models, interest to adopt or willingness to buy autonomous vehicles for two wheeled vehicle users is mostly influenced by perceived trust and risk rather than perceived concerns/benefits for middle and old age groups in India. Perceived ease of use has the most significant marginal effects on two-wheeler riders' perceived risks towards autonomous vehicle adoption, which accounted for more than 20 % of unexplained variance. Results from this research may offer insightful diversity of attitudes towards adoption of autonomous vehicle technologies in India through the means of increased efficiency and reduced travel time and costs along with riders’ risks towards privacy and safety.

## Highlights

• Riders' perceived risks and intention to use autonomous vehicles were explored under Indian disordered traffic scenario.
• Perceived risks of TW riders decreases with increased perceived usefulness and trust of AV technology.
• Young female two-wheeler users propel 10 times more towards four-wheeler autonomous driving.
• Riders have 0.9 times less influence on the intention to use (ITU) autonomous vehicles compared to public transport captive users for shopping-based trips.

## Graphical abstract

**Figure:**
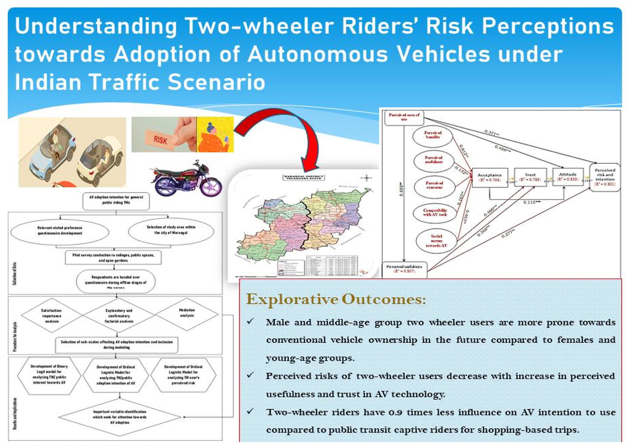

## 1 Introduction

The global status report on road safety in the year 2022 released by the Ministry of Road Transport and Highways [1] conveys that about 1.36 million people die every year in road crashes. In India, about 4, 49,002 road accidents are being reported, among which 1, 51,113 are deaths and 4, 51,361 injuries [2]. Developing countries like India face crash severity rates (crash severity rates are based on accident severity indices (ASI), which depend on the weighted summation of fatal, serious, and minor injuries during accidents) of near to 15–18 % (for the year 2019, leading to a potential ASI value of over 103), and therefore, there is an incessant need for a safe and reliable accident prevention assessment. Starting in the 1970s, studies [[2], [3], [4]] indicate human errors like failure, fatigue, impairment, and distraction contribute over ninety percent of accidents worldwide. Recent developments in vehicle technology have deployed autonomous vehicles (AVs) for testing on public roads [5,6]. Autonomous vehicles have the potential to compensate for human errors while driving, which reduces congestion, emissions, and fuel consumption compared to public transit (like BRTS or MRTS) [7]. According to the McKinsey Centre for Future Mobility (2019), the market output value for driverless cars and autonomous driving for the year 2030 within global and regional levels will be expanding to whopping 1.6 trillion US dollars (14.98 million Euros), which is almost two times the total revenue generated by leading car manufacturers like Ford, General Motors, Toyota, and Volkswagen for the year 2017. Recent studies [8,9] indicate an increase in the autonomous vehicle (AV) penetration rate of 75 percent may reduce 95 percent of road accidents. This also allows people who are incapable to drive car/two-wheeler to travel by car, keeping in mind their limitations [10]. Driving skills play major roles in traffic collisions, and driver stimulus, as well as interpreting the situation during collisions as perceiving the situation, acts as an intellectual as well as emotional factor to avoid a collision by making the appropriate decision of decelerating or braking. These autonomous vehicles (AVs), otherwise called self-driving vehicles, can reduce or eliminating human error based on the vehicle class adopted, from 0 (non-automated) to 5 (fully automated), depending on how much human resource is important to control a vehicle [11,12].

The Society of Automotive Engineers (SAE) has classified autonomous vehicles into six categories for assessing levels of autonomy, from no automated features (level-0), driver assistance (level-1), partial automation (level-2), conditional automation (level-3), high automation (level-4) and full automation (level-5) [12]. Motorized or powered two-wheelers (TW), such as motorcycles, scooters, or e-bikes, have become increasingly popular in developing nations [[13], [14], [15]]. According to recent statistical studies [16,17], about 29 percent of road fatalities in the world are related to motorized two-wheeler and bicycle users. In low- or middle-income countries (LMIC) like India, Taiwan, the Philippines, and Vietnam, daily commuters and the residential population frequently rely on two-wheeler for short-distance trips (for shopping or work).

**Fig. 1:**
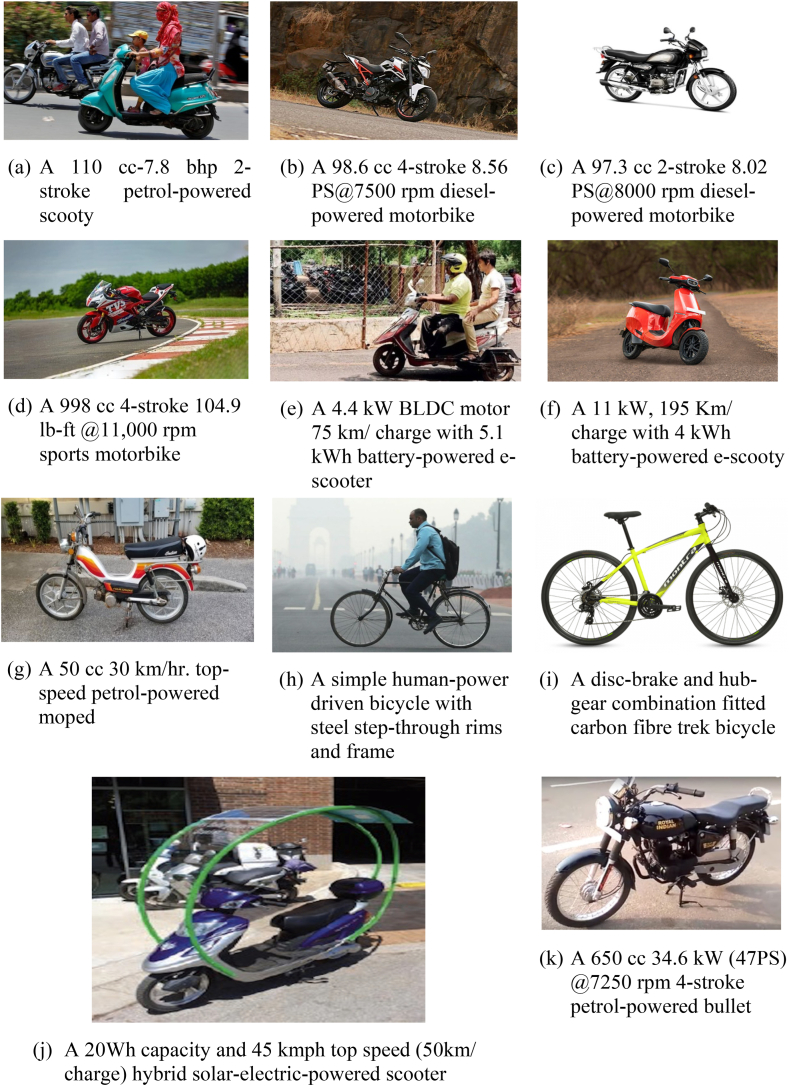
Illustration of two-wheeler vehicle categories operational on Indian roads.

According to recent estimates by Ref. [18], in India, around 160-million-kilometre trips are being shared, primarily by two-wheeler riders, owing to a yearly vehicle growth rate of more than 10 %. Around 41 % of private vehicle users in India and about 75 % in Thailand use motorized and non-motorized two-wheelers for home-based and recreational trips [19]. Motorized (fuel/battery/electric powered) and non-motorized (human pedalled) two-wheeled vehicles commonly operating on Indian roads are shown in Fig. 1. In this study, opinions from riders using the above-mentioned scooter, moped, and motorcycle categories have been utilized while those using stand-up scooters (which are prevalent in European and Latin American countries) have been neglected. Hence, research concerning motorized two-wheeler safety and the adoption intention of two-wheeler riders towards autonomous vehicles would be of immense help in deciding safety strategies for traffic managers and also for the general public. In developing countries, studies on AVs are less explored, and studying the public's perception of the intention to adopt AVs or two-wheeler riders' risk perceptions of AVs might be difficult due to the non-availability of such vehicles in developing countries. It is important to understand the existing methodological framework for such new technology adoption intentions by the public. Emerging technologies such as autonomous vehicles hold significant potential for integration into mixed traffic systems in the near future. Therefore, a clear understanding of AV usage perception regarding the adoption intention of these vehicles will play a crucial role in multi-modal sustainable integration. Although these technological advancements are going forward, AV crashes (like in February 2016 or in May 2016), as reported by Ref. [20], are still rampant. Researchers such as [21] have made strong assertions that naturalistic driving data should be collected for optimizing these driving systems, suggesting 1000 AVs traversing for about 50 years with over 15 billion kilometres of naturalistic driving data for optimization. Self-driving vehicles have not yet officially entered the Indian market even after deployment periods between 2021 and 2022 in most of the European and East Asian nations [[22], [23], [24]]. But, a few prototypes are presently being tested in Tier-I cities like Bengaluru and Mumbai, like the camera-sensor-based zPod car marketed by Minus Zero Robotics Private Limited [23] and the autonomous vehicle deployment platform by the Automotive Research Association of India-ARAI (https://cms.araiindia.com/MediaFiles/ARAIAutonomousVehicleDeploymentPlatform_1303.pdf) both of which were introduced in late 2020s. Both of these prototypes are still under testing on probing grounds (across the country), have not yet entered the connected vehicle environment, and only have Level-1 (cruise control) and Level-2 (which can perform partial steering and acceleration adjustments) categories of autonomous operations. But unfortunately, during the testing of all these prototypes, drivers still need their hands to steer the wheel. Therefore, during such transition periods of autonomous vehicle commercial field applications on roads of lower-middle income economies like India, the authors have collected data for conditional (Level-1/Level-2) AV implementation using offline questionnaires (using both expert-opinions and traveller intercept surveys).

(Source: [18])

Using stated preference face to face two-wheeler rider perceptions from Telangana, the present study aims to understand and study the user perceived risks of adopting or purchasing four-wheeler autonomous vehicles (AV) in India. About 1673 experts and general two-wheeler rider public opinions out of 1800 interviewed two-wheeler riders from five districts of Telangana (in India) were randomly sampled. Conventional technology adoption models like technology acceptance (TAM), theory of planned behavior (TPB), unified theory of technology acceptance and use of technology (UTAUT) and theory of innovation diffusion (IDT) [[25], [26], [27]] were tested using the 1673 opinions for Indian scenario. The three-fold objectives of the study are:

1. To identify TPB/TAM/UTAUT/IDT constructs affecting two-wheeler riders' perception in adopting/purchasing four-wheeler autonomous vehicles in India.
2. To propose mixed TAM-TPB-UTAUT-IDT model for understanding two-wheeler riders' willingness to shift towards four-wheeled autonomous vehicles in India.
3. To rank TPB/TAM/UTAUT/IDT constructs based on Indian two-wheeler rider autonomous vehicle technology adoption intentions and perceived risks.

This section presents background of the research and motivation behind such a study. Rest of the paper is organized as follows: Section 2 gives a general description of previous AV adoption intention-based studies and problem statement. Section 3 provides methodological framework of the research. Section 4 depicts discussions and proactive results from the analysis presented through structural equation (SEM) and random forest (RF) feature selection followed for validating the TPB, TAM and UTAUT concepts for two-wheeler opinions. Section 5 displays pragmatic benefits of conducting this study while Section 6 briefs about certain limitations besides conventional normative benefits of this research. Finally, Section 7 presents concluding statements about findings, contributions and scope for future research.

## 2 State-of-art on general public and vulnerable road user perceptions regarding autonomous vehicles (AVs) usage

### 2.1 Outlook of the past studies on autonomous vehicles and two-wheeler perceived risks on AV adoption

Bibliometric analysis (co-citation, coupling based on countries, documents and authors) was conducted in biblioshiny web-app (integrated in bibliometrix package in R-studio) on web-of-science (WoS) and Scopus database after formulating keyword combinations like “autonomous vehicle” OR “automated vehicles” OR “driverless vehicles” AND “adoption” OR “intention to use” OR “perceived risks” AND “mixed traffic” OR “mix* traffic*” AND “two wheelers” OR “powered two-wheelers*”. Literature search between years 2004–2024 yielded 2035 journal articles and conference proceedings which were then filtered (using exclusion criteria) to 786 useful documents on public perception studies on autonomous vehicle adoption/buying. It is pivotal to mention here that none of the 1054 studies dealt with two-wheeler users’ perception on adoption intention/perceived risks of purchasing or willingness to shift to autonomous vehicles. The bibliometric analysis: co-occurrence of the keywords has been carried out using VoS viewer software.

**Fig. 2:**
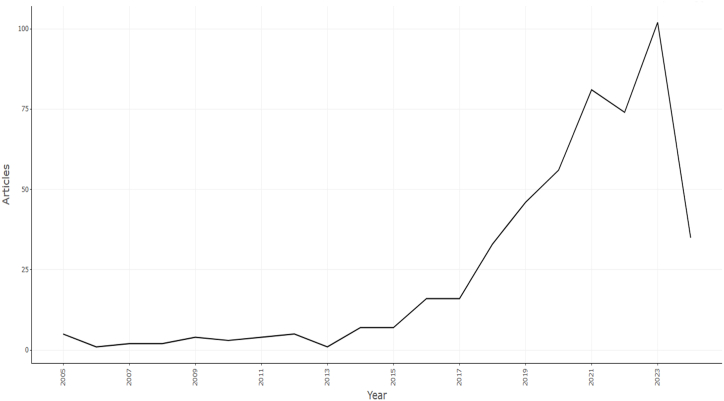
Annual scientific production of articles on autonomous vehicle adoption.

**Fig. 3:**
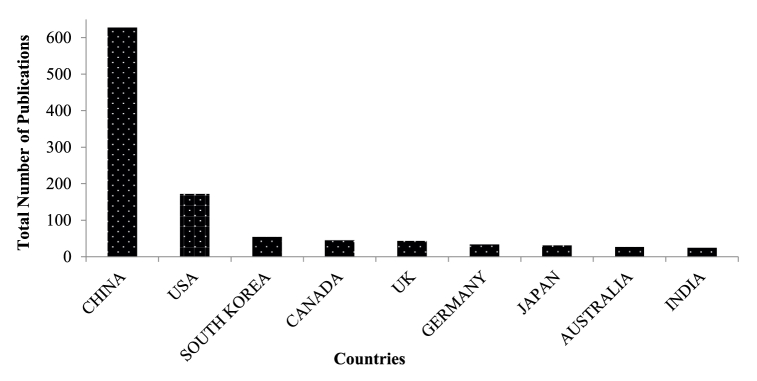
Top 10 countries working on autonomous vehicle adoption intention research between 2004 and 2024.

The authors investigated about 765 keywords, but only 35 of them met the threshold when we set the minimum number of occurrences for a keyword at ten. Keywords mostly associated with adoption intention are perception, attitudes, safety, acceptance, compatibility, risks, etc. Investigating previous studies may yield a wealth of research exploring public perceptions about AVs and the factors that affect their intention to use, willingness to pay, acceptance of this technology, and other perceptions such as perceived attitudes, trust, usefulness, etc. Fig. 2 shows a declining trend of annual scientific contribution (from the year 2004–2024) of about 78 countries (having low, middle and high-income distributions) to understand public perceptions of autonomous vehicle adoption intention. Additionally, Fig. 3 shows India has lesser than 10 publications (within the time frame of 2004–2024) on autonomous vehicle adoption intention research among top 10 countries with China leading with 678 publications followed by USA with 174 publications. Hence, auxiliary research on utility of technology acceptance (TAM), theory of planned behavior (TPB), unified theory of technology acceptance and use of technology (UTAUT) and theory of innovation diffusion (IDT) are necessary to understand behavioural risks and intention to use autonomous vehicles in India. According to Fig. 3, China, USA and Europe hold about more than 80 % of studies on autonomous vehicles as validated from bibliometric literature reviews conducted by Ref. [23]. Developing (low or middle) economies having greater proportions of powered two-wheeler proportions operating under non-lane based traffic flow environment lacks sufficient research on autonomous vehicle as ascertained through detailed prior bibliometric and meta-analytic reviews by Refs. [24,28]. Cyclists and motorcyclists both constitute about 60 % of vulnerable road users, along with the disabled, elderly, and children (pedestrians), depending on their capabilities. Past studies also suggest that motorcyclist fatality rates are 20–45 times higher than those of car drivers for 1 km of distance travelled [29,30]. Bicycle and two-wheeler riders share road space with conventional and automated vehicles (ADAS-supported vehicles in India). Therefore, their risk perception might be quite high compared to pedestrians, as supported by the large number of their collisions due to human errors [16]. Pammer et al. [31] found that two-wheeler riders (like motorcyclists and bicyclists) are more likely to adopt autonomous vehicles because of important factors like the detection of vulnerable road users (VRUs) and automated prioritization of various calibrated driving actions. But there are fewer studies like the former, and therefore further investigation into the mitigation of two-wheeler crash risks with the hypothetical introduction of autonomous vehicles is a need of the hour.

**Fig. 4:**
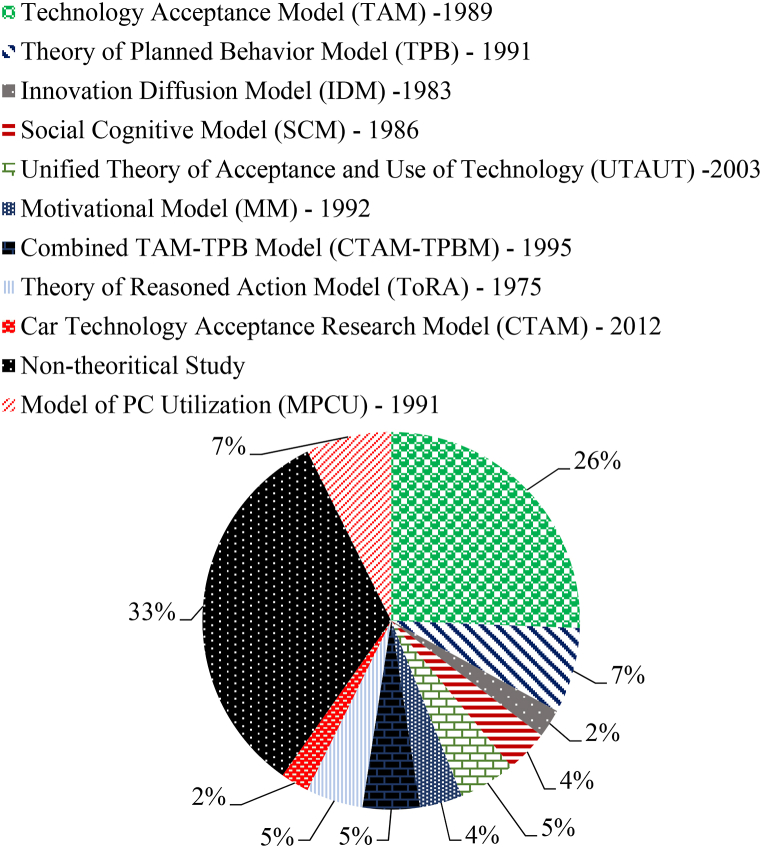
Summary of theoretical psychological models used worldwide for understanding autonomous vehicle adoption intentions (*Based on literature available till April 2024*).

Most of the recent studies on psychological parameters affecting public's acceptance towards autonomous vehicle technology have been identified based on a rigorous literature search. The technology acceptance behaviour of drivers can help associate certain potential psychological factors affecting AV acceptance. About 87 relevant literatures relating to technology acceptance model (TAM), theory of planned behaviour (TPB), theory of innovation diffusion (IDT), unified theory of acceptance and use of technology (UTAUT), theory of reasoned action (ToRA) and other theoretical models most widely used are summarized through Fig. 4. Most of the studies [28,32,33] have used behavioural intention as dependent parameter affecting public acceptance of AVs. The TAM uses perceived usefulness (PU) and perceived ease of use (PEOU) as explanatories and behavioural intention as dependent variable for understanding public attitudes towards AV acceptance [33,34]. The TPB uses attitude towards behaviour (ATI), subjective or normative beliefs (SN) and perceived behavioural control (PBC) as independent constructs and behavioural intention as dependent variable to understand public's AV acceptance behaviour [33,34]. The UTAUT theory assumes that intention to use technology depends upon effort expectancy (analogous to perceived ease of use), performance expectancy (analogous to perceived usefulness), social influence (analogous to subjective norms) and facilitating conditions (individual's judgement on supporting infrastructure and environmental conditions) [32,33].

**Fig. 5:**
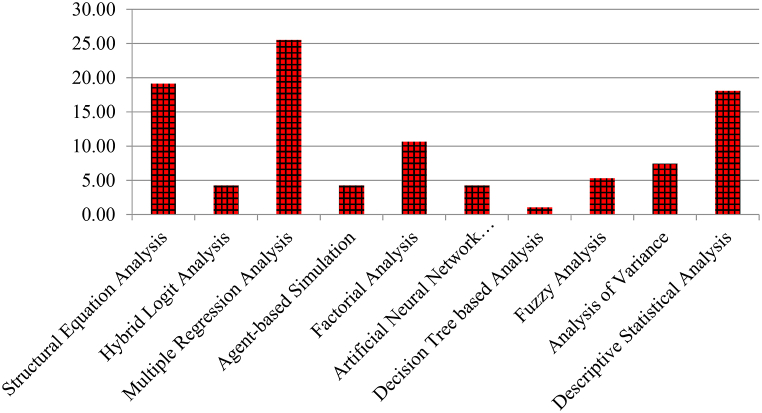
Summary of autonomous vehicle acceptance model analysis methods conducted between 2000 and 2024 (*Based on literature available till April 2024*).

Theory of innovation diffusion (IDT) on the other hand assumes any person's adoption decision will be influenced by compatibility (consistency of technology with individual needs and experiences), triability (exposure to technology analogous to acceptance), observability (influencing trust), relative advantage (analogous to perceived benefits), image (analogous to cultural reputation), and complexity (analogous to perceived concerns) [25,34,35]. The ToRA (often termed as extended TPB) uses attitudes, subjective norms as independent variables and behavioural intention as dependent variable for understanding public's AV behavioural usage [34,36]. No such prevalent research combining all four theories (TAM, TPB, UTAUT and IDT) have been conducted to the authors' knowledge till date under Indian mixed traffic conditions. In this study, for the first time, a hybrid TPB-TAM-UTAUT-IDT theoretical conceptual AV behavioural intention model was tested under Indian scenario considering two-wheeler rider opinions. Fig. 5 shows the research methods utilized for analysing psychological theoretical concepts used in all the 87 relevant literature.

As observed multiple regression analysis, structural equation models and descriptive statistical assay dominate while least used methods are decision tree-based models and hybrid logit analysis methods. Ziakopoulos et al. [27] used random forest (which is a decision tree derived unsupervised learning technique) for clustering attributes affecting AV acceptance. The researchers also pointed the need to combine SEM outputs as inputs to random forest feature search algorithm towards ranking TPB/TAM/UTAUT constructs. Therefore, in this study the authors have combined SEM and RF to achieve the purpose of explaining autonomous driving acceptance more accurately. Table 1 lists a few crucial perception-based studies conducted in high and middle-income economies across the world to understand public risks and intention to use autonomous vehicles between 2014 and 2024.

**Table 1:** Previous studies of behavioural adoption intention of autonomous vehicles.

Authors	Year of the Research	Country	Model Used	Objective/Inferences
Choi and Ji [14]	2015	South Korea	Single Fold Structural Equation Model	Based on 552 drivers SP data, technical competence, system transparency and situation management have positive effect on drivers' trust in AV technology
Bansal et al. [10]	2016	United States of America	Multinomial Logit Model	To forecast long term impacts of connected and shared autonomous vehicles
Hohenberger et al. [37]	2016	Germany	Conceptual Moderated Mediation Models	Willingness to use autonomous vehicles in Germany
Buckley et al. [38]	2018	United States of America	Driver simulator study	Significant predictors of intention to use AV are attitude, perceived behavioural control and subjective norms
Hulse et al. [39]	2017	United Kingdom	Multinomial Logistic Model	Assessed road users' perception towards acceptance of AVs based on safety
Madigan et al. [40]	2016	United States of America	Game Theory	Interaction between pedestrians and autonomous vehicles were analysed
Panagiotopoulos and Dimitrakopoulos [41]	2017	United Kingdom	Structural Equation Models	Perceived ease of use has lesser impact on AV adoption while perceived usefulness is more preferential towards AV behavioural intention to use
Webb et al. [42]	2018	Australia	Multinomial Logit Model	The authors measured residents' willingness towards trading off internal combustion engine vehicles for shared electric AVs Higher income commuters and married couples are more likely to shift to AVs
Pettigrew et al. [43]	2018	Australia	Stated Preference Choice Experiment & Latent Profile Analysis	The authors highlighted importance of public education and awareness of AVs for resolving fear levels and enhance trust and control issues based on 1, 624 responses
Stoiber and Hoerler [44]	2019	Switzerland	Exploratory Regression Analysis	Online choice opinions from 709 participants regarding automated cars, automated pooled-use taxis and automated public transport shuttles were collected. Automated cars and automated public transport are more preferred models among Switzerland residents
Yuen et al. [45]	2019	China	Confirmatory Factorial Analysis & Structural Equation Modeling	The innovation diffusion theory, perceived value theory and trust theory was introduced in this study. Perceived usefulness and ease of using AVs have positive influence on behavioural intention to use
Yuen et al. [34]	2020	Vietnam	Structural Equation Models	268 participant data were collected to assess adoption of shared autonomous vehicles (SAV) Theory of planned behavior constructs like attitude, subject norm, perceived behavioural control are effective predictors of SAV adoption in Vietnam
Parsa et al. [46]	2020	Chicago (United States of America)	Machine Learning Models like K-nearest neighbours, Random Forest and eXtreme Gradient boosting	Impacts of 0 % and 100 % penetration rates of connected vehicles on annual daily traffic of Chicago POLARIS data set using ML techniques were assessed. Gross population density has direct impact on Annual daily traffic under 100 % CAV penetration rates Demographics, transport and land use characteristics does not affect ADT under AV penetrations
Amiri et al. [47]	2021	Canada	CFA and SEM analysis	Logistic or supply chain firms with high e-commerce sales may adopt AVs in Canada
Bakioglu et al. [48]	2022	Turkey	Multinomial Logit and Mixed Logit Model	323 web-based responses were collected to assess parameters like liability issues, cost, safety and environmental issues Effects of partial and full automation were assessed on private (PV), shared (SV) and ride-hailing (RV) vehicles People's ride share preference increases with increased cost reduction due to shared ride.
Zhang et al. [26]	2022	China	Congruence Path Model and Response Surface Analysis	Effects of congruency between individual expectations and perceptions towards utilitarian and hedonic motivation to adopt AVs were assessed based on 877 responses. Factors relevant for adopting AVs in China are safety, uniqueness, enjoyment and reputation. Hedonic motivation (i.e., enjoyment) mainly drives public's behavioural intention to use AVs rather than utilitarian needs (like willingness to pay).
Ziakopoulos et al. [27]	2023	Greece	Self-organizing maps and random forest (RF) feature selection	Most important factors affecting AV adoption in Greece are distance limitations of AVs and replacement of AI navigator in understanding driver conscience & principles in AVs Respondents were separated into two clusters according to SOM analysis RF classified 78 % of tested datasets correctly

### 2.2 Inferences from previous studies and problem statement

There are research gaps in the form of attributes affecting commuters' decisions to adopt autonomous vehicles (AVs) and how they feel about the risks of doing so in situations where traffic flow isn't smooth. Additionally, investigations on special dimensions of classes for different predictors and perceptions of two-wheeler riders are also less explored. According to the former sub-section 2.1, various factors like attitude, trust, and perceived usefulness are some of the most important drivers of AV adoption intention [49]. Studies such as [49,50] also shared driving experience on AV usage trust as a contingent parameter. Further research by Ref. [50] propagates the willingness to place importance on AV technology. Hence, public attitudes and behavioural intentions for AV technology adoption need to be studied rigorously [49,50]. Some of the studies discussed in this section also deal with the performance evaluation of autonomous vehicles (AV) using agent-based simulation strategies. On the other hand, the concept of autonomous vehicles and naturalistic field operating tests (other than the floating car method) is still under jurisdiction in developing countries like India and has not yet been employed to understand virtual heterogeneous driving behaviour. In the context of lower middle-income economies and regions with a gross national income per capita of $1136 to $4465 (like India, Bangladesh, Morocco, the Philippines, Iran, and Vietnam) and upper-middle-income economies and regions with a gross national income per capita of $4466 to $13,845 (like Indonesia, Mexico, Thailand, South Africa, and Paraguay) it is quite necessary to explore attitudinal factors towards AV adoption intention and perceived risks towards AV technology (as suggested by Refs. [51,52]) under mixed traffic conditions. Studies have less explored on the effects of factors like mobility behaviour, mode preferences, and land use characteristics [3,49] on AV adoption intention, which need to be addressed for better AV policy implementation. Sankeerthana and Kadali [22] recommend that India should focus on public awareness schemes impelling Indian drivers to use autonomous vehicles after their formal market penetration soon. Some of the possible causes of autonomous vehicle circumvention in low/middle income (developing) economies are lack of efficient road infrastructure, lack of public awareness about autonomous vehicle technology, amassed share of joblessness, high degrees of lane-indiscipline, absence of appropriate road signage and markings and poor technology up gradation [[22], [23], [24]]. Other pivotal behavioral factors affecting AV avoidance is price and unemployment [25] in lower-middle and low income economies/regions within East-Asia Pacific like India, Bangladesh, Pakistan, Sri Lanka, Bhutan, Nepal. Additionally, AV prototypes developed for Indian roads rely mostly on advanced driving assistance (ADAS) operations and detection. However, ADAS operations are based upon proper and serviceable road markings/signage which seems to be out of place in majority of metropolitan urban areas within India. Additionally, varying two-wheeler penetration rates (a merely, 41 two-wheeler per km for the state of Kerala to a huge, 637 two-wheeler per km for the city of Surat) through the entire diverse geography of India [18] makes AV application too much complex in the present scenario. The indulgence of cognitive factors (users' actual perception) and personality traits (users' expectations) and their inter-dependence have not yet been tested under heterogeneous traffic mixes [26]. Therefore, through this study, the authors want to provide sound theoretical basis to mitigate the difference between two-wheeler riders' actual perceptions and expectations towards autonomous driving on Indian roads through a path-based probabilistic approach using stated preference questionnaire.

## 3 Methodology followed for this research

**Fig. 6:**
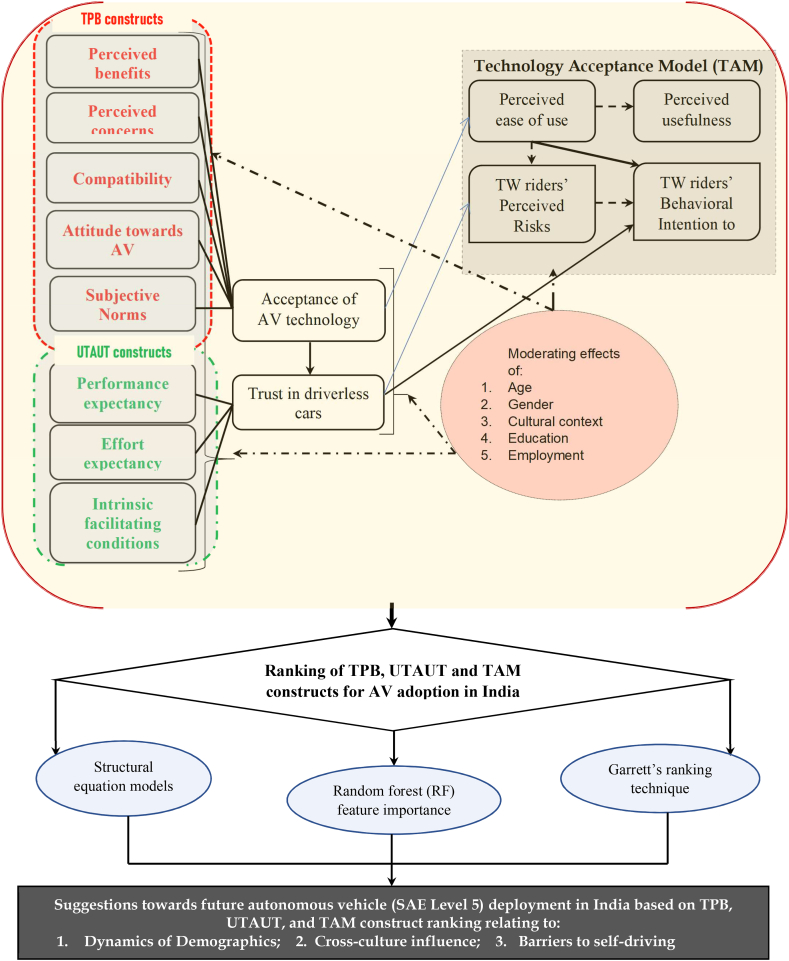
Conceptual framework of the research (Note: TPB – theory of planned behaviour; UTAUT – unified theory of acceptance and use of technology, AV – shared/independent autonomous four-wheeler vehicles; SAE- Society of Automotive Engineers).

Fig. 6 shows the proposed overall conceptual framework of the present study. A minimum of 11–21 interviews were conducted with the offline questionnaire [53,54]. The qualitative method was fixated on gaining insights to evaluate the multifaceted nature of public opinions. Intention for understanding subject-oriented perspectives using a more structured layout was performed. The study involves the identification of public perceptions towards AV adoption and the calculation of the risk associated with AVs when mixed with two-wheeler in general. As these AVs are not yet operational on Indian roadways even in the approaching 23rd century, public and two-wheeler rider perception-based responses were collected to get a clear picture of the probable inclusion of AVs in the approaching century. Sample AV videos were shown to the public and two-wheeler riders who are not accustomed to this technology and an on-road and simulated experiment was also conducted. Factors considered after investigation and sensitivity analysis concerning perception weightages are attitude (ATI), trust (TRU), acceptance (ACC), risky behaviour (PR), benefits or concerns (B/C), and intention to use (ITU). The research aims to narrow the gap in AV adoption in Indian districts using the extended hybrid aggregated technology acceptance (TAM), theory of planned behaviour (TPB), unified theory of acceptance and use of technology (UTAUT) and innovation diffusion (IDT) principle. This framework as depicted through Fig. 6 encompasses India's unique socio-cultural and geographical features. The methodology also offers a more comprehensive understanding of four-wheeled AV adoption by vulnerable two-wheeled users within middle income developing South Asian countries like India. Suggestions enthralling India's fully automated AV deployment soon in terms of dynamics of demographics, district specific cross-culture influence and barriers in self-driving form imperative benefits of this research work. This in turn may instigate global conversation on autonomous vehicle transport technologies.

### 3.1 Stated preference survey locations and survey sampling strategy

Telangana state is situated in the southern corner of the Indian peninsula and has an average land coverage of 1, 12, 077 square kilometres with a population of 3, 50, 03, 674 (according to 2011 Census). According to the recently published reports by the Government of Telangana Transport Department, the state has an average registration of more than 10 million two-wheeler vehicles at the end of the financial year 2022–2023. Table 2 depicts a summarization of the five districts chosen for conducting the 21 state choice interviews for understanding AV adoption intention and perceived risks among two-wheeler riders in India.

**Table 2:** Smart and Atal Mission for Rejuvenation and Urban Transformation (AMRUT) cities in Telangana (Ministry of Urban Development, 2016; AMRUT, 2016) selected for SP surveys.

Rank	City Name	District	Type of Municipal Framework	Population (as per 2011 Census)	Status
**1**	Hyderabad	Hyderabad	Greater municipal corporation	6, 809, 970	Smart city
**2**	Warangal	Warangal (Urban)	Greater municipal corporation	8,11, 844	Smart city
**3**	Nizamabad	Nizamabad	Municipal corporation	3, 10, 467	AMRUT
**4**	Karimnagar	Karimnagar	Municipal corporation	2, 60, 889	AMRUT
**5**	Khammam	Khammam	Municipal corporation	1, 84, 252	AMRUT

**Fig. 7:**
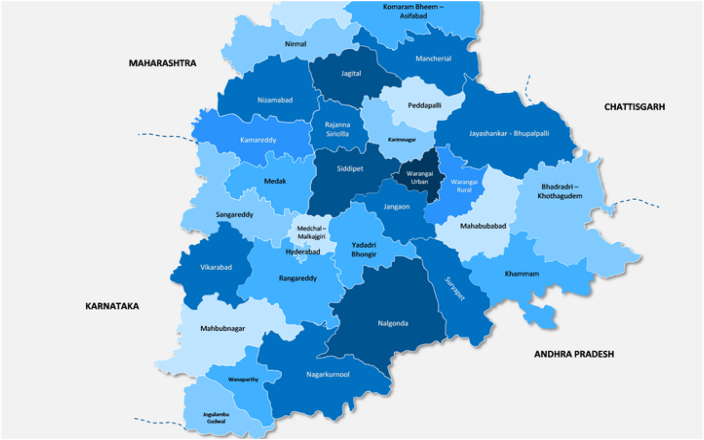
District wise Division of Warangal, Telangana State (Source: https://warangal.telangana.gov.in/map-of-district/).

Various universities, public open spaces, district roads, outer ring roads, bus stops, intersections and public bike sharing docking stations located within urban and sub-urban areas of Hyderabad, Warangal, Nizamabad, Karimnagar and Khammam districts within the state of Telangana, India were selected for conducting stated preference surveys from December 2021 to February 2022 (Mondays to Fridays between 11 a.m. and 4 p.m.). A D-efficient SP survey design strategy [48] was adopted for minimizing standard error of the constructs estimated. Randomized design of stated choice experiments according to (Design of Experiments via Random Design 2021) was followed. Every single respondent were presented with 4 scenarios and every scenario were properly explained to all 1800 respondents initially identified from the five districts of Telangana. The number of samples for the study is based on Cochran's formula [55], and an error of 3 % was considered for 0.005 significance levels. The district-wise division of Warangal city in the state of Telangana is depicted in Fig. 7. Auto rickshaws and two-wheelers are the most preferred modes of transportation within the sub-urban and CBDs of the trio cities. In, the sample size guestimate formula using z-score scaling as depicted in Equation (1), $S_{\mathrm{Min}}$ corresponds to the minimum required number of samples for a population size of P while, ME is the error margin corresponding to significance, *sig* value of 0.05 with a z-score (z) of ± 1.96.

(1) $$S_{\mathrm{Min}}=\frac{\frac{z^{2}\times sig\left(1-sig\right)}{{ME}^{2}}}{1+\left(\frac{z^{2}\times sig\left(1-sig\right)}{{ME}^{2}\times P}\right)}$$

In this study, stratified samples were adopted for the survey design to ensure an equal distribution of males and females, while maintaining diversity in age groups. We also selected participants from diverse socio-demographic and educational backgrounds. 1673 responses were effectively collected using SP interviews (corresponding to required minimum of 1529). Frequency estimation of the TAM/TPB/UTAUT/IDT constructs and research findings was construed based on the several indicators and all 50 questions were identified from the data. Interviews (N = 21) are organized in a semi-structured manner, based on predefined guided and open-ended questions (attached as supplementary material) that take about 10 min on an average. First section of the questionnaire introduces the experts and the two-wheeler rider respondents to the state-of-the-art thought process whether current two-wheeler riding population will shift towards four wheeled autonomous vehicles (AV) while plying through urban roads (under prevailing signage and road marking conditions) in India. The importance of conducting such a study was explained through accident statistics from previous MORTH [1] reports. Advantages and disadvantages of self-driving vehicles focusing on utilitarian (necessity-based) and hedonic (enjoyment-based) motivations towards their adoption under Indian mixed traffic scenario were thoroughly explained by conducting weekly tutorial classes (about 1 h) over a span of 2 months in the year 2021. All the seven enumerators selected for collection of the SP questionnaire data were trained about different AV technology through tutorial sessions of 1 h every week throughout the months of July to September 2022 through online sessions (due to COVID 19 Second phase pandemic outbreak). Description about all six automation levels (Level-1 to Level-6) as per Society of Automotive Engineers (SAE) were thoroughly explained to all the respondents through 3–4 min of short, customized video clips made in the laboratory. Description of the sensors and camera setups within the in-vehicle orientation (https://www.araiindia.com/services/technology-and-products/autonomous-vehicle-deployment-platform) including concepts of LIDAR (Light Detection and Ranging), RADAR (Radio Detection and Ranging), GNSS (Global Navigation Satellite Systems) and IMU (Inertial Measurement Unit) were also thoroughly taught to both the respondents as well as the enumerators in collaboration with expert talks from well-known AV developers in India like Swaayatt Robots (https://www.swaayattrobots.com/), Minus Zero (https://www.minuszero.ai/). Additionally, certain on-road Indian AV test run videos were played in front of the experts and the two-wheeler riders to make them familiar with Indian on-road hover applications under different terrains (like rolling, plain, hilly with extreme rough pavement condition) as can be observed from the following video clips of AV test runs:

1. https://www.youtube.com/shorts/9bvJAudyoPs
2. https://www.youtube.com/watch?v=nQ9oxrL1LE4
3. https://www.linkedin.com/posts/sanjeevsharmaiitr_autonomousdriving-autonomousvehicles-deeplearning-activity-7154708431604482048-1OfS/
4. https://www.swaayattrobots.com/research/
5. https://www.minuszero.ai/technology

**Fig. 8:**
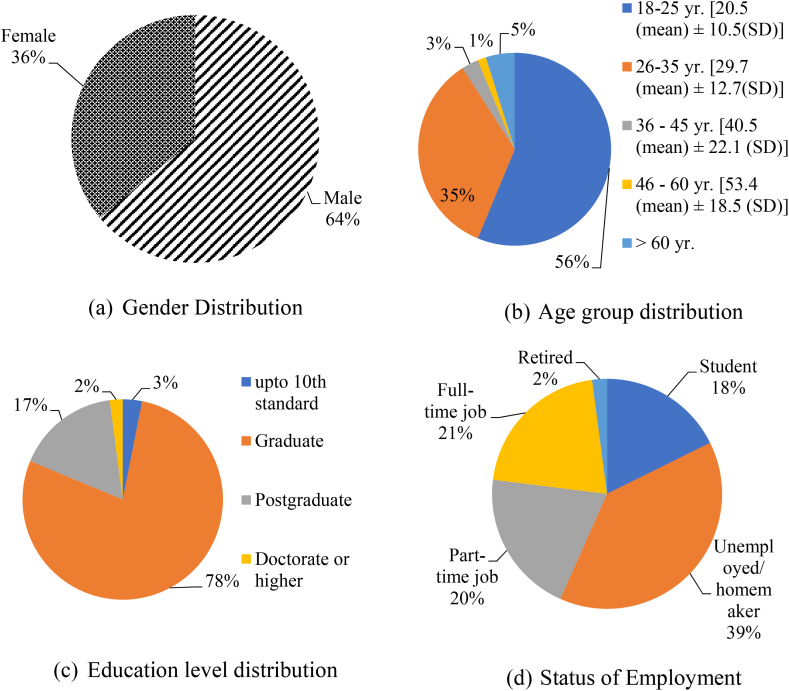

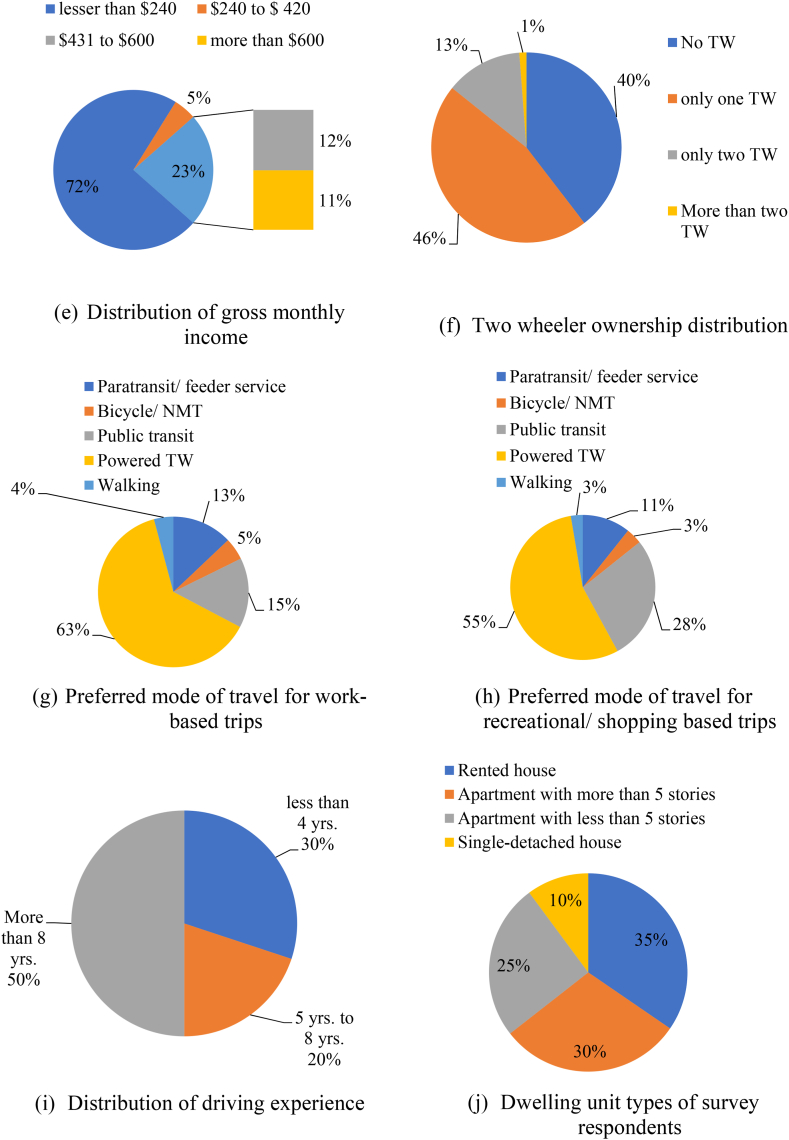

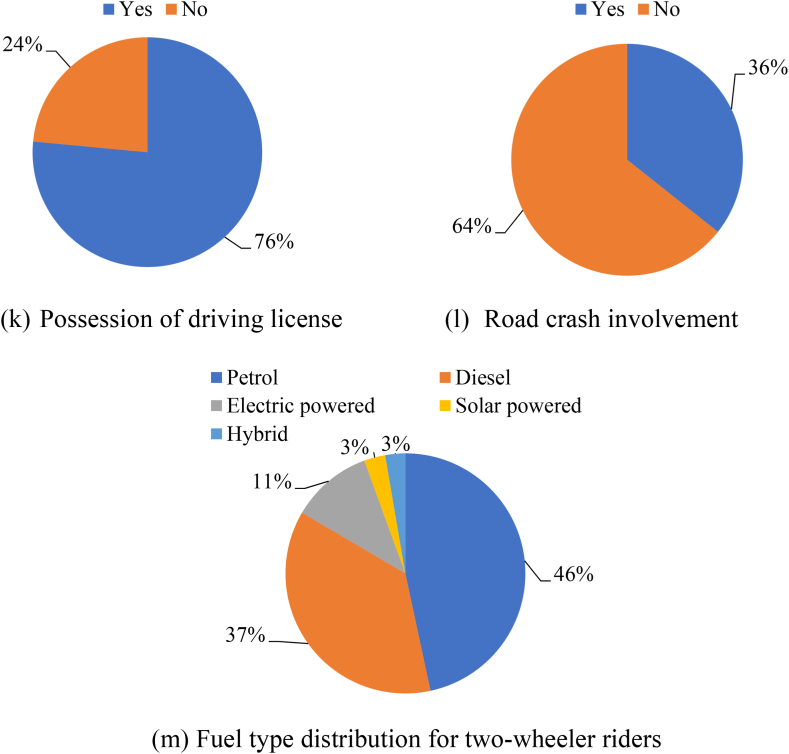
Summary of collected responses for AV Adoption Intention from experts and two-wheeler riders within Telangana State of India

**Fig. 9:**
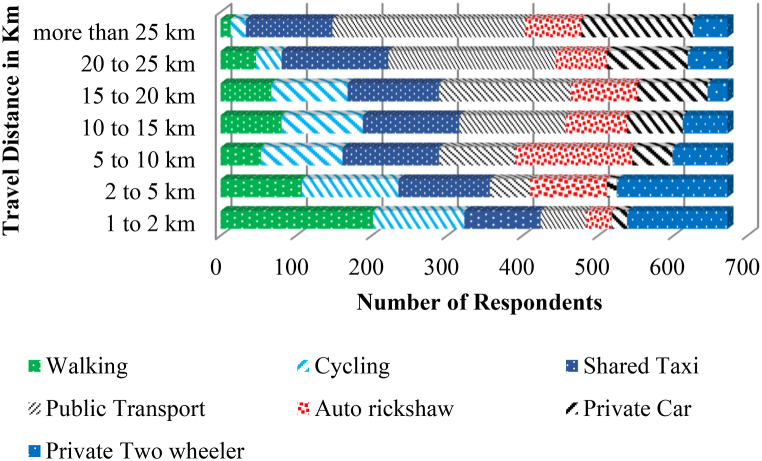
SP survey respondent preferred mode of travel for varying travel distances.

Fig. 8 summarizes distribution of demographic and socio-economic factors (age, gender, monthly income, education level, employment status, private vehicle ownership, and dwelling unit type) and travel behaviour related (like mode of travel to work/recreation, road crash involvement, etc.) attributes of all 1673 SP survey respondents. The descriptive statistics of the expert and two-wheeler rider characteristics (which forms Section 2) of the questionnaire mostly describes about gender, age groups (18–25, 26 to 35, 36 to 45, 46 to 60 and above 60 years), education levels (up to 12th class, graduate, post-graduate and doctorate or higher), levels and type of employment, monthly income (with levels being lesser than Rs. 20,000 ($240), Rs. 20,000 ($240) to Rs. 35,000 ($430), Rs. 36,000 ($431) to Rs. 50,000 ($600) and Above Rs. 50,000 or $600), number of two-wheelers ownership and preferred mode of travel during shopping and work-based trips, frequency of trips by two-wheelers, age of the two-wheeler currently under usage. The results reveal that 64 % of the experts and 36 % of the two-wheeler rider respondents were male leaving the rest as female ones. According to the MORTH [1] regulations the gender ratio as mentioned above for both the respondent categories were found to be within specified ranges. Section 3 of the questionnaire comprised of 50 numbers of open-ended questions with binary options of agreement/disagreement using five point Likert scale. It can be inferred from Fig. 9 that non-motorized mode of transportation (i.e., walking and cycling) is preferred mostly by city dwellers for short range trips. But as distance increases, private vehicle mode share increases. Public residents in AMRUT cities (like Karimnagar, Khammam and Nizamabad) prefer busses as public transits instead of auto rickshaws or shared taxis compared to smart cities like Warangal and Hyderabad. People using two-wheelers in metropolitan city like Hyderabad prefer shared two-wheeler taxis even for longer distances (5–10 km) as it is easy for them to use a smartphone compared to residents of Karimnagar, Khammam, and Nizamabad. For further distances (20–25 km) people in AMRUT cities like Karimnagar, Khammam and Nizamabad rely either on private cars or on public buses. But due to heavy congestion in smart cities like Hyderabad and Warangal people mostly prefer private two-wheelers, prepaid ride sharing taxis (two-wheelers) and auto rickshaws over public bus rapid transits (BRT) or mass rapid transits (metro rail) to avoid heavy congestion problems during peak hours.

### 3.2 Theoretical framework of the study

Renowned psychological theories like technology acceptance (TAM), theory of planned behaviour, unified theory of technology acceptance (UTAUT) and innovation diffusion (IDT) principle were combined in this research for understanding risks, concerns and intention of two-wheeler riders' towards purchasing four-wheeled autonomous vehicles. Moderation and mediation analyses were performed on path analysis hypothesis using structural equation models (SEM). Feature ranking of socio-economic, demographic, travel behaviour and two-wheeler psychological behaviour constructs is also performed using random forest (RF) algorithm. Identification of best TPB/TAM/UTAUT/IDT parameter corresponding to AV adoption use behaviour derived from the SEM and RF models was done using Garrett's ranking criteria. In the context of theory of planned behaviour (TPB), perceived behavioural control (PBC) is completely different from a person's intention and depends on the importance of a person's difficulty with his or her vehicle riding capabilities. Both PBC-1 and PBC-2 help weigh a two-wheeler's difficulty against their capability of having the intention to ride AVs instead of a conventional (petrol/diesel powered) two-wheeler. Perceived risks (PR) generally account for any loss incurred by a two-wheeler user in terms of physical injury, leakage of privacy, or in financial form. Intention to use, on the other hand is a subjective probability measure of two-wheeler riders' decision-making capability towards AV adoption under emergency or complex behavioural and jurisdictional situations. Perceived usefulness (PU) on the other hand takes into consideration two-wheeler riders' assessments of goal achievement during daily routine tasks. Fig. 8 shows that in India people use private powered two-wheelers for short distance trips (up to 5 km) and public transport for long distance trips covering more than 25 km for daily commute to work. Perceived benefits (PC) include attributes like time and cost savings, environmental sustainability, accessibility, and mobility enhancement. Innovation diffusion (IDT) constructs like attitude towards AV adoption behaviour (ATI), trust in AV technology (TRUT), and compatibility with self-driving vehicles (COMP) vary from rider to rider and are often characterized by vulnerability and uncertainty. Factors considered along with their items, constructs, and statements are illustrated in Table 3. These are taken from various studies after exploring previous literature searches. A five-point Likert scale is adopted (strongly disagree, disagree, neutral, agree, and strongly agree) for all these factors. Perception-gathering factors are subjected to basic descriptive statistics. All average ratings are from neutral to agree for all the factors except two-wheeler riders' violations and errors. We utilized the responses during the satisfaction-importance (IS) strategic analysis to rank the most influential parameters that influence the adoption of AV among two-wheeler riders. The respondents were asked the following questions based on the following TAM, TPB, and IDT constructs:

1. Intention to use autonomous vehicles under Indian traffic (ITU)
2. Perceived usefulness for AV technology adoption by public (PU)
3. Perceived ease of use for autonomous vehicles by public (PEOU)
4. General public's trust towards AV technology (TRUST)
5. Two-wheeler riders' attitudes towards adoption of AV under Indian mixed traffic (ATI)
6. Two-wheeler riders' perceived risk towards AV adoption (PR)
7. Perceived Behavioral Control of public for autonomous vehicles (PBC)
8. Subjective norms of two-wheeler riders while buying autonomous cars (SN)
9. Two-wheeler riders' compatibility with AV technology (CMP)
10. Two-wheeler riders' acceptance towards driving/riding autonomous vehicles (ACC)
11. Perceived benefits on using autonomous vehicles under mixed traffic conditions (PB)
12. Perceived concerns regarding AV adoption by public (PC)

**Table 3:** Several constructs or items or statements corresponding to identified TPB/UTAUT/IDT factors included in the two-wheeler rider AV adoption behaviour questionnaire.

TPB/TAM/UTAUT/IDT Factors	Main Constructs and Levels/Statements
Intention to use AV (ITU)	**I am willing to use AVs:**
ITU-1	In place of conventional cars
ITU-2	For future transportation on Indian roads
ITU-3	Depending upon recommendation from recent AV users
ITU-4	If provided access to use such products in future
ITU-5	Because I think these products are unique
ITU-6	I will use AV provided I have idea about the maintenance costs
ITU-7	Knowing that I would spend more time in work/household chores
Perceived Usefulness (PU)	**I will use AV as they:**
PU-1	AV will increase my driving performance
PU-2	Allowance for cognitive distraction like watching movies, listening to music, be on cell phones during AV trips
PU-3	Helps reduce accidents
PU-4	Helps in driver impairment (drowsy, drinking, drugs, etc.)
PU-5	Helpful when I am not physically fit to drive
Acceptance (ACC)	**How likely I am to use AV:**
ACC-1	Lack of driving skills won't let me drive
ACC-2	Physical health condition make it difficult for me to drive in future
ACC-3	For short range trips (like groceries, pharmacies, etc.)
ACC-4	Long distance travelling
Perceived ease of use (PEOU)	
PEOU-1	AV will enhance my driving skills
PEOU-2	Easiness and comfort ability while AV control my ride
PEOU-3	AV interaction would require greater mental effort
Trust (TRUT)	
TRUT-1	AVs are efficient than human driven vehicles
TRUT-2	My family will be safe driving in AVs
TRUT-3	Governmental safety policies will make AV adoption easy for me
TRUT-4	Reliability of AVs are more and can trust them
TRUT-5	AVs can make safe decisions during emergencies
Attitude (ATI) Behavioural beliefs and outcome evaluations of AV adoption:	
ATI-1	Autonomous cars will enhance my personality
ATI-2	I think AVs are safer than powered two-wheelers
ATI-3	I would like to try new technology in automobile kinesis
ATI-4	If driving AVs make me happy, I will buy these for my daily use
Perceived Behavioral Control (PBC)	
PBC-1	It is easy for me to drive a petrol/diesel/electric TW rather than driving AV
PBC-2	It is easy for me to maintain an AV compared to my current conventional TW
PBC-3	I want to know detailed operational features of an AV
Compatibility (CMP)	**I think AVs will be compatible for future Indian traffic conditions:**
CMP-1	Current Indian infrastructure and technology supports AV adoption
CMP-2	AVs are compatible universally under all traffic flow regimes
CMP-3	AVs will easily fit my driving regime and need
Perceived Risks (PR)	**I will be adopting AVs even if:**
PR-1	AVs don't perform well or create problems
PR-2	AVs create financial losses
PR-3	AVs hamper my privacy
PR-4	I need to share road space with conventional fuel powered two-wheelers
Subjective Norms (SN)	**Social normative beliefs will influence my AV adoption:**
SN-1	Purchasing branded AVs will distinguish people in a positive manner
SN-2	Purchasing branded AVs will make me feel superior among my peers
SN-3	My friends and family would like me to buy an AV in future
SN-4	Purchasing branded AVs will help me earn more reputation
Perceived Benefits (PB) I believe the following benefits can materialize after I buy an AV:	
PB-1	Lower vehicular emissions compared to conventional vehicles
PB-2	Increased fuel economy compared to my current private vehicle
PB-3	Lower vehicle insurance rates
PB-4	Lesser travel time and travel cost during regular commute to work/home
PB-5	Fewer crashes and decreased accident rates
PB-6	More enjoyment (like watching movies, playing games or browsing social media)
PB-7	Better emergency/incident mitigation due to improved artificial intelligence tools
PB-8	Lesser traffic congestion and service delay on high-speed arterials
PB-9	Better psychological satisfaction due to improved travel efficiency
Perceived concerns (PC)	**I will not shift towards automated driving because of concerns like:**
PC-1	Absence of separate control rooms for guiding driverless vehicles
PC-2	Increased loss of human driver jobs in India
PC-3	Absence of proper regulatory framework in tier-II and tier-III cities
PC-4	Compromised vehicle security due to hacking
PC-5	Data privacy leakage during location and destination tracking
PC-6	Increased crash propensity with non-motorized two-wheeler users
PC-7	Adverse effects of bad weather on smooth AV operations during peak hours
PC-8	High maintenance and charging costs
PC-9	Lack of proper signage and signal coordination infrastructure in Indian cities

The construction of the path analysis models for this study is based on 20 proposed hypotheses. All the 20 hypotheses were based on theoretical and empirical context of India's technical and cultural environment. With reference to the theory of planned behavior (TPB), sub-scales selected include attitude towards AVs, social norms for AV adoption in India, and perceived behavioral control for understanding risk perceptions (perceived risk) towards adoption intentions of AVs. Previous studies have adopted perceived usefulness (PU) and perceived ease of use (PEOU) from the technological acceptance model (TAM) as inputs into the AMOS software. Following are the hypotheses considered in this research while developing the path model structures:

**Table:** 

Hypothesis 1:	Perceived usefulness has positive effects on trust towards AV technology
Hypothesis 2:	**Perceived usefulness** has positive effects on AVs **perceived risks** towards AV technology
Hypothesis 3:	**Perceived ease of use** has positive effects on **perceived usefulness**
Hypothesis 4:	**Perceived ease of use** has positive effects on **attitude** towards AV technology
Hypothesis 5:	**Perceived ease of use** has positive effects on **perceived risks** towards AV technology
Hypothesis 6:	**Perceived benefits** has positive effects on **acceptance** towards AVs
Hypothesis 7:	**Trust** has negative effects on **Perceived Risks** of using AV technology
Hypothesis 8:	**Perceived concerns** has negative effects on AV **acceptance** for two wheeler riders
Hypothesis 9:	Two wheeler rider **Compatibility** with AVs has positive effects on AV **acceptance**
Hypothesis 10:	**Subjective norms** followed by two-wheeler riders will improve **trust** in AV technology
Hypothesis 11:	AV technology **acceptance** has positive effects on **trust** in AVs for two wheeler riders
Hypothesis 12:	**Acceptance** of two wheeler riders towards AVs has positive effects on **attitude** towards AVs
Hypothesis 13:	Two wheeler riders' **trust** in AVs has positive effects on rider **attitudes**
Hypothesis 14:	Riders' cultural **Attitude** has positively influence on **Perceived risks**
Hypothesis 15:	Riders' **Perceived Risks** have negative effects on Behavioural **Intention to Use**
Hypothesis 16:	**Perceived ease of use** positively affects riders' **Intention to buy/use** an AV
Hypothesis 17:	**Perceived usefulness** of AVs will enhance riders' **Intention to use**
Hypothesis 18:	**Performance expectancy** has positive effects on **Intention to use**
Hypothesis 19:	**Effort expectancy** will have positive effects on Indian two-wheeler drivers AV adoption
Hypothesis 20:	Better intrinsic **Facilitating conditions** positively affects two-wheeler drivers' **Attitudes**

**Fig. 10:**
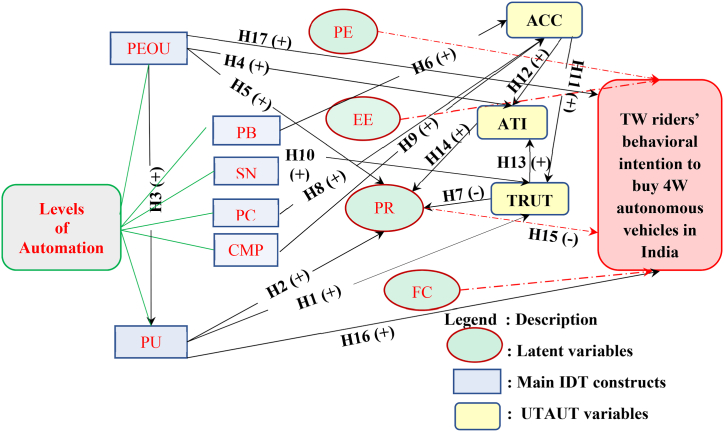
Behavioural conceptual model in SEM analysis followed for assessing two-wheeler riders' AV adoption intention in India Note: PEOU: Perceived ease of use; PU: Perceived usefulness; PB: Perceived benefits; SN: Subjective norms; PC: Perceived concerns; CMP: Compatibility with technology; PE: Performance expectancy; EE: Effort expectancy; PR: Perceived risks of using AV technology; FC: Intrinsic facilitating conditions; ACC: Acceptance; ATI: Attitudes towards behavioural intention to use AV; TRUT: Trust in driverless cars; TPB: Theory of planned behavior; UTAUT: Unified theory of acceptance and use of technology; H: Hypotheses.

Fig. 10 shows the typical theoretical conceptual model framework for the SEM hypotheses assumed in this study. The proposed behavioural conceptual model includes adoption intention of four-wheeled autonomous vehicles by two-wheeled riders' as the dependent variable while TPB constructs like PEOU, PB, SN, PC, CMP and PU while TAM/TPB/UTAUT constructs like ACC, ATI and TRUT along with ITU are used as independent variables. Several innovation diffusion theory variables like performance expectancy (PE), effort expectancy (EE), perceived risks (PR) and intrinsic facilitating conditions (FC) latent variable effects on two-wheeler behavioural adoption intention to buy AVs were also tested based on moderation of demographic characteristics like age and gender. While two-wheeler driving experience, gross monthly income was utilized for testing internal consistency of riders’ responses during SEM analysis. Some of the latent dependent variables and sub-questions are.

### 3.3 Analysis methods for understanding two wheeler riders’ perceived risks and intentions to adopt autonomous vehicles in India

Explanatory Factorial Analysis (EFA), Confirmatory Factorial Analysis (CFA) and Structural Equation Modeling (SEM).

Explanatory factorial analysis (EFA) is used to perform an initial investigation on the data for the identification of patterns in relatively large item sets. The preliminary aim of this tool is to uncover the underlying structure or relationships between measured variables. Kaiser-Meyer-Olkin (KMO) and Bartlett's test of sphericity are the primary sources of judgments while conducting factorial analysis. As an integral part of EFA, Bartlett's test of sphericity tests the validity and proposition of observed responses to be included during modelling. On the other hand, confirmatory factorial analysis (CFA) tests the hypothesis already existing in various studies. Researchers often adopt the hypothesis, which describes the relations between measured variables and hidden or latent variables, after gaining sufficient knowledge about previous literature. CMIN/DF (preferred value of 3), comparative FIR index (CFI >0.9 is preferred), standardized root mean square residual (SRMR <0.08, preferably), and root mean square error of approximation (RSMEA <0.08, preferably) are common endurance parameters that help assess CFA fitness. Simultaneously, Cronbach's alpha (α) and Guttmann's reliability (Lambda-4) are utilized for assessing the internal consistency of sub-scales during CFA. This study also employs mediation analysis, which frequently aids in determining the causal sequence of a dependent variable based on a mediating variable through the actions of an antecedent variable. This implies that a third variable creates the mediation effect by intervening between the effects of the other two. Now, if the relationship between the two latent variables is quite significant and remains unaffected, mediation is unsupported. On the contrary, if the effect of the two latent constructs is significant and unchanged, partial mediation exists. There is full mediation in cases where the relationship or effect between both latent variables is statistically insignificant. Utilizing such a strategy, as expressed in the former statements, public perception about AV adoption along with perceived risk by two-wheeler riders under Indian traffic flow conditions will become easier to investigate.

Structural equation modeling (SEM) is commonly attributed to the multi-argument and multivariate method, which is universally used for hypothesis testing to understand pivotal relations among interacting variables from a response-based survey. Both path diagrams and matrices can be utilized for evaluating SEM, with the latter expressing the relations in a graphical manner using fewer conventions. The rectangle (□) and circle (○) represent the observed and latent variable respectively while arrow (→) shows the relations during prediction. The arrows can be defined as matrices using Equation (2):

(2) Y=A+B.Y+C.X+E

In Equation (2), Y represents a 1X P matrix including endogenous variables; X is a 1 X Q matrix resenting exogenous variables; B is a P X P matrix consisting of path coefficients relating endogenous variables; C is a P X Q matrix consisting of mediation coefficients relating endogenous variables to the exogenous variables, while E being a 1 X P matrix representing random error exponents, with P and Q being the number of endogenous and exogenous variables, respectively; and A is the model constant or intercept.

Internal consistency of the clustered variables to evaluate degree to which all test items within the hypotheses listed above produce significant results were assessed using Cronbach's alpha computed using Equation (3) in which, N is the number of estimated parameters, $\sigma_{j}$ variance corresponding to jth component of the sample and $\sigma_{T}$ is to variance corresponding to total test scores for the same sample:

(3) $$C-\alpha =\frac{N}{N-1}\left(1-\frac{\sum_{g=1}^{k}\sigma_{j}^{2}}{\sigma_{T}^{2}}\right)$$

Goodness of fit (GFI) of CFA/SEM analysis can be judged easily from the following parameters which are often checked for model accuracy:

**Table:** 

$\chi^{2}$ –value:	This statistic is used for testing in-between sample inconsistency and covariance within the models. The value of this statistic increases with sample size and number of variables.
**Tucker-Lewis Index (TLI):**	It is an incremental fit index widely utilized for linear mean and structure modelling during explanatory factorial analysis. Values greater than 0.90 is appreciable.
**Comparative fit index (CFI):**	This index generates a direct connection with model fit and an independent model and always displays the latent variables compared to indicators. Values greater than 0.91 is appreciable
**Root mean square error of approximation (RSEA):**	Considered as the most beneficial index and helps estimate parameters used for fitting the final population covariance matrix. Values lesser than 0.1 are considered best for SEM analysis.
**Standardized root mean square residual (SRMR):**	SRMR is calculated after dividing model fit residuals with standard error of the residual and solves most of the problems created by RSEA. Values lesser than 0.1 are appreciable.

#### 3.3.1 Random forests (RF)

**Fig. 11:**
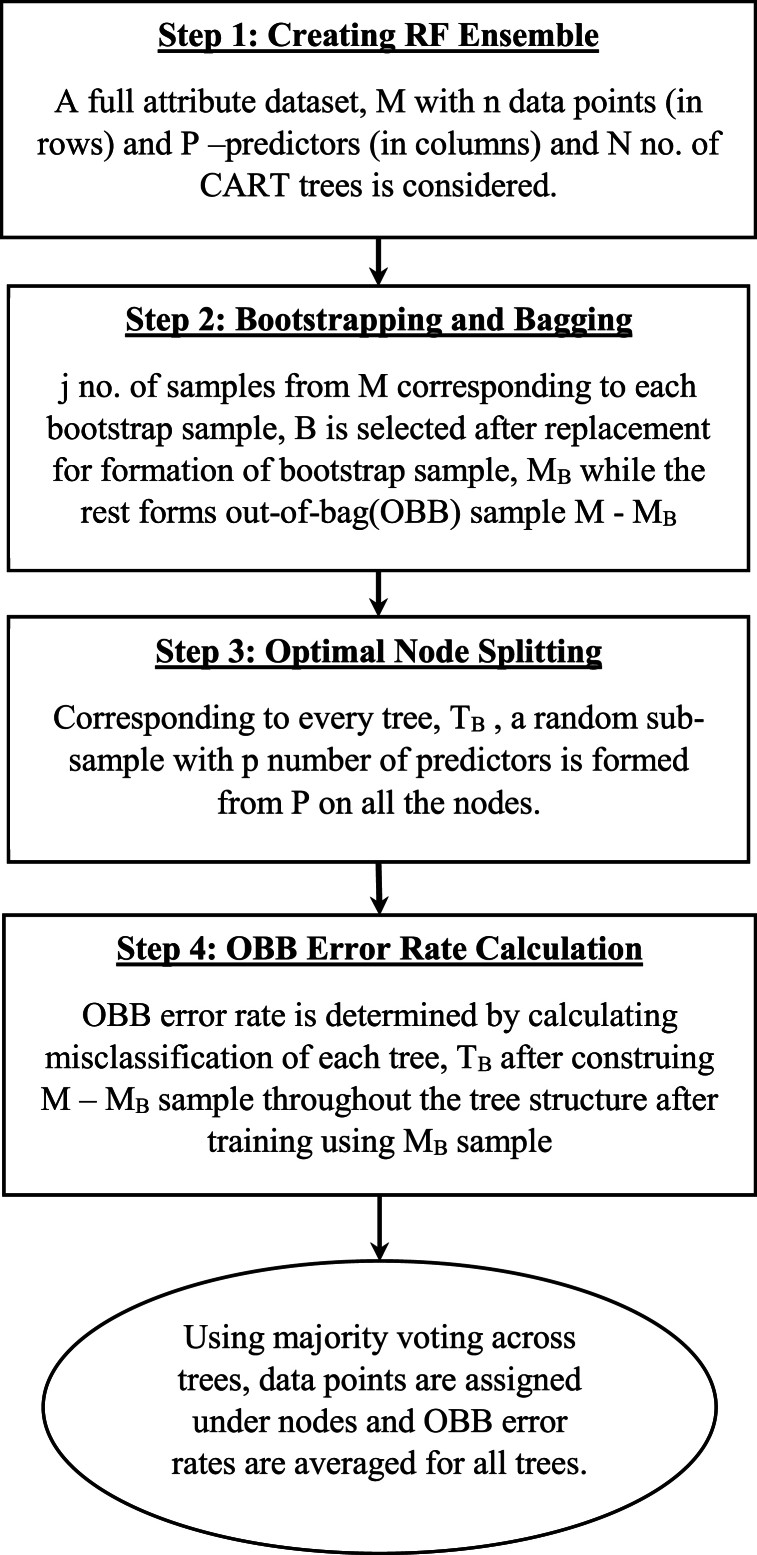
Process flow diagram showing random forest algorithm followed for this study.

Random forest (RF) regressive strategy for TPB/TAM/UTAUT/IDT hypothesis testing on collected two-wheeler response as shown in the flowchart (Fig. 11) has been followed for the present study. Random forest (RF) method helps integrate bootstrapping and feature selection. It is an attribute specific ensemble technique which is majorly utilized for classification and regression [27,56]. According to Refs. [27,57], RF algorithms are often used in transportation studies for finding attribute importance and they are capable of overcoming limitations of many statistical and competing machine learning techniques. The random forest algorithm gathers votes from hundreds of classification and regression trees and then overlays them to congregate to a regression outcome or a decision corresponding to the most probable class.

Breiman [58] illustrated this algorithm after introducing the concept of bootstrap sampling and bagging. During repeated random sampling, some of the data may appear more than once and the rest may be left out within the bootstrap sample which is also known as out-of-bag error (OBB). Therefore, random feature selection is often introduced during bootstrapping process. According to Guo et al. [59], random forest can be utilized during stable selection of important components. The development of decision trees which forms background of RF can be illustrated with following simple steps:

1. Target variable and attribute variable determination with data concentration within root nodes before splitting.
2. Variable dataset splitting into child nodes using suitable splitting algorithm (for example, ID3 splitting) on the explanatory variables.
3. Then, every child node is treated as a parent node for additional splitting within the RF recursive process.

The diversity within the decision trees created using RF displays two levels of randomness – (i) Diversified dataset of variables with constant sample size; (ii) diversified set of attribute variables for splitting every parent node. RF is capable of picking variability within the data through strong technical components [60]. Random forests’ predictability depends upon – (i) In-between base tree correlation which needs to be reduced; (ii) Tree performance which needs to be strengthened; (iii) Number of trees which should be large concerning computational efficiency. Therefore, in RF, three pivotal features need calibration namely, no. of trees or the forest size (N), no. of splitting attribute variables (M) and maximum tree depth (D). A diversified set of observations are bootstrapped for every decision tree in RF. Additionally, entrant splitting attributes within each tree are selected using random feature selection from an entire set of explanatory variables. Until and unless every tree attains their maximum depth splitting process is continued. After, application of majority voting strategy, outcome predicted is the most probable predicted within that ensemble. MeanDecreaseGini based on the Gini impurity index is utilized in RF for split calculation [60]. The importance measure for a specific attribute is achieved based upon average decrease of Gini impurity index for all trees generated in the forest. Corresponding to an entrant splitting attribute, A_i_ with categories C_1_, C_2_ … C_J_, the Gini impurity value is determined using Equation (4).

(4) $$G\left(A_{i}\right)=\sum_{j=1}^{J}P\left(Y_{i}=C_{J}\right)(1-P\left(Y_{i}=C_{j}\right)=1-\sum_{j}^{J}{P\left(Y_{i}=C_{j}\right)}^{2}$$

In Equation (4), J denotes total number of classes, $P\left(Y_{i}=C_{J}\right)$ represents the estimated categories within attribute, $Y_{i}$ = $C_{J}$ probabilities. In our study, random forest with bagging and random sub-space strategy is utilized as a predictive formulation to increase the prediction accuracy. Assessment of the RF prediction is done using metrics like accuracy (ACCU), sensitivity (SENSE), specificity (SPEC), through receiver operator curve analysis. Accuracy relates to overall model's prediction accuracy in predicting feature importance. Sensitivity is used for evaluating models' predictive power in guessing positive classes when actual outcome is positive while specificity evaluates a model predictive power in correctly guessing the negative classes when actual outcome is negative.

## 4 Results, analysis and discussions

The general public's perception towards the adoption of fully automated vehicles (AVs) is analysed in this research using binary and ordinal logistic regression models. We analyse the questionnaire survey results using importance-satisfaction rating and ranking methods, which are based on the formulation of the items and constructs presented in Table 3. Confirmatory factorial and path analysis were performed on the survey data results for the identification of important explanatory parameters affecting two-wheeler riders' perceived risks towards AV technology. Both EFA and CFA are performed using the lavaan latent growth templates [25] in SmartPLS software (version 4.0.1), which is quite user-friendly considering its unique covariance-based filtering abilities. The path and mediation analysis were performed in the AMOS (IBM SPSS) software. The binary logit and ordinal logistic regression models were performed in R-studio (version 4.3.1) using polr command within the MASS package using the following steps:

**Table:** 

**Step 1: Loading of libraries within the R-workspace:**
library (foreign)
library (MASS)
**Step 2: Reading of data and running analysis using polr:**
dat < - read.dta(“TW-PR.txt”)
m < - polr (apply ∼"pr”, data = dat)
summary (m)
**Step 3: Combining odds ratio within its confidence intervals using cbind command and storing confidence interval within object ci:**
(ci < -confint(m))
**Step 4: Binding the transpose of the ci object with coef (m) and exponentiation of the coefficients:**
exp (cbind(coef(m), t(ci))

### 4.1 Two-wheeler rider respondents

Responses from more than 1800 individuals were gathered. Based on heterotrait-monotrait discriminant validity tests only 1673 responses were found suitable for assessing AV adoption intention of two-wheeler users. About 30 min were invested for every respondent to answer the questions after repeated tutorial sessions of 1 h/week being conducted regularly between July and September 2021. The responses were collected in-field at a convenient place and time according to their voluntariness. The respondents were shown video clips and video demonstrations of Indian autonomous vehicle ZPod (SAE Level-5) pilot run tests. All possible missing values (like “I don't want to disclose” – kind of responses to questions 1 to 16, 23 to 35 as shown in Table B1 of Annexure B in Appendix section) were deleted as per principal component analysis. The average age of two-wheeler rider respondents was 36 years 7 months (standard deviation of 12 years 10 months). About more than 70 % of the respondents (both experts and two-wheeler riders) belong to the middle age group between 36 and 45 years. It was also observed that among the random sampling done from the population of respondents about more than 85 % of the experts and 87 % of the two-wheeler riders had valid driving license with average driving experience between 10 and 20 years and they all had vehicle insurance in case of emergencies.

### 4.2 Discussions on attitudinal question ranking results

The results from normalization and standardization of the respondent filled scores reveal that most of the two-wheeler riders’ rate maintenance costs to be more detrimental in determining AV adoption more than journey or fuel costs. Also, most of the experts agree with the preference that AVs can improve their travel time and cost for long distance trips. Also, many experts and two-wheeler riders agree that the current Indian road infrastructure and leakage of privacy policies would hinder their AV adoption rates in future. About 45 % of the experts and more than 76 % of the two-wheeler riders agree that it is easy to make safer gap acceptance decisions with their conventional fuel powered two wheelers rather that allow any artificial intelligent agent to make their choice of gap acceptance/rejection under Indian mixed traffic conditions.

**Fig. 12:**
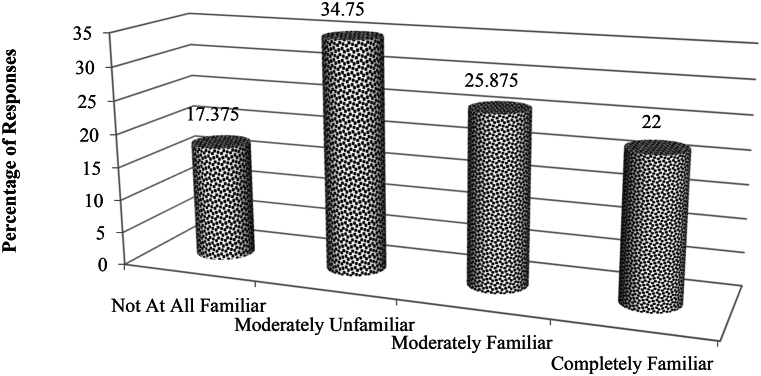
SP survey respondent familiarity towards autonomous vehicle technology.

Most of the respondents also agreed that adverse weather conditions in some parts of India (like Jammu, Kashmir, Leh & Ladakh, Assam, Nagaland, etc.) will make AV technology transfer weak. All the respondents agreed that AV technology applications on Indian roads will make vulnerable road users (pedestrians and non-motorized riders) feel safer in taking gap acceptance decisions. Around 76 % of the experts and merely 45 % of the two-wheeler respondent agreed to the fact that driverless two wheelers will reduce journey travel time by 50 % compared to conventional powered two wheelers. Fig. 12 portrays familiarity of the 1800 random respondents towards autonomous vehicle technology. Majority of the respondents (more than 30 %) who participated in the 21 stated preference interviews were moderately unfamiliar with AV technology and use of AVs for passenger mobility. Although more than 20 % of the respondents did have complete idea about AV automation levels under mixed traffic conditions. Perceived behavioural control is derived from the concept of “theory of planned behaviour,” in which we measure the extent to which people believe that they can complete a specific task. Table 4 summarizes the means and standard deviations of all variables considered during this study. We filtered the questionnaire survey responses from two-wheeler riders for errors and excluded missing data. Therefore, the data included during modelling is free from any errors. Further, an ANOVA test was conducted to find the impact of the demographic variables on AV intention adoption and on perceived risk, as shown in Table 5. According to the test results depicted in Table 6, education level, transportation mode utilized for work or during shopping, and interest in using AVs all have a significant impact on both the intention to use and the perceived risk of the public or two-wheeler riders in using AVs. Education levels (with F-statistic = 6.508 for p < 0.001) and mode of transportation utilized during shopping trips (F-statistic = 3.258 for p < 0.001) have a broader outlook on the adoption interest of AVs (F-statistic = 22.481 for p < 0.001) towards two-wheeler conventional drivers.

**Table 4:** Descriptive of the data collected using questionnaire survey.

Sub-scales	Mean	Standard deviation
**Intention to use (ITU)**	3.535	0.882
**Perceived usefulness (PU)**	3.517	0.920
**Perceived ease of use (PEOU)**	3.451	0.927
**Trust (TRU)**	3.409	0.909
**Attitude (ATI)**	3.465	0.932
**Perceived Risk (PR)**	3.179	0.897
**Perceived Behavioural Control (PBC)**	3.356	0.899
**Subjective Norms (SN)**	3.330	0.873
**Compatibility (COMP)**	3.120	0.981
**Acceptance (ACC)**	3.410	0.880
**Perceived benefits (PB)**	3.489	0.886
**Perceived concerns (PC)**	3.438	0.884

**Table 5:** ANOVA Results for Intention to use and perceived risk of the public towards AV adoption.

Dependent variable	Demographics	F-statistic	[df1, df2]	Significance values
**Intention to use (ITU)**	Gender	1.136*	[1, 672]	0.287
	Age	3.428**	[4, 668]	0.009
	Education level	6.717**	[4, 668]	0.000
	Employment	1.154**	[5, 667]	0.330
	Level of Income	3.891**	[3, 669]	0.009
	Work purpose mode	3.831**	[6, 666]	0.001
	Shopping purpose mode	6.849**	[6, 666]	0.000
	Possession of Licence	0.048**	[1, 672]	0.827
	Driving experience	0.783*	[4, 668]	0.536
	Familiarity with AVs	133.181**	[1, 672]	0.000
**Perceived Risks (PR)**	Gender	1.647**	[1, 672]	0.200
	Age	0.989*	[4, 668]	0.413
	Education level	6.508**	[4, 668]	0.000
	Employment	1.56*	[5, 667]	0.169
	Level of Income	3.046*	[3, 671]	0.028
	Work purpose mode	3.439**	[6, 666]	0.002
	Shopping purpose mode	3.528**	[6, 666]	0.002
	Possession of Licence	1.24**	[1, 672]	0.825
	Driving experience	0.815*	[4, 668]	0.515
	Familiarity with AVs	22.481**	[1, 672]	0.000

Note: *p < 0.05, **p < 0.001.

**Table 6:** ANOVA Results on the two wheeler rider's perceived risk towards AV adoption.

Dependent variable	Demographics	F-statistic	[df1, df2]	Significance values
**Two wheeler rider's perceived risks (PR)**	Gender	0.891**	[1, 672]	0.346
	Age	1.913*	[4, 668]	0.106
	Education Levels	4.634**	[4, 668]	0.000
	Employment	2.182*	[5, 667]	0.054
	Level of Income	0.783*	[3, 671]	0.503
	Work purpose mode	1.816**	[6, 666]	0.093
	Shopping purpose mode	4.716**	[6, 666]	0.000
	Past accident effects	2.139**	[1, 671]	0.144
	Interest in AVs	0.005**	[1, 671]	0.941

Note: *p < 0.05, **p < 0.001.

**Fig. 13:**
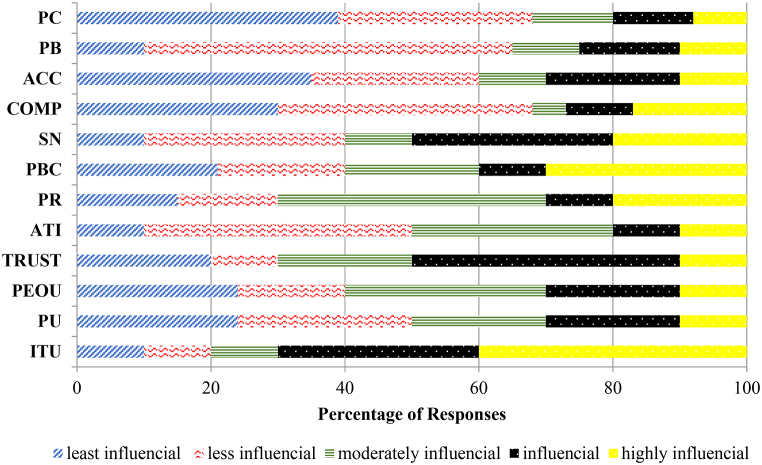
Two-wheeler opinion distribution on TAM/TPB/UTAUT/IDT factors.

There is an inherent lack of understanding of two-wheeler riders or bicycle riders’ perceptions of their intention to use AVs, which has propelled the authors in this present study to use both empirical and inductive tactics for qualitative analysis of data. Ranking allotment according to satisfaction, importance percentage, and IS ratings is depicted in Table 7. Fig. 13 shows a distribution of percentage two-wheeler respondent opinions on TAM/TPB/UTAUT/IDT indicators based on all the 21 stated choice interviews in the five districts of Telangana. The empirical tactic employed for analysing the data and to filter severances and connections among the several sub-scales categorized under intention to use and perceived risk of two-wheeler riders. The inductive tactic [61] was comprehended with the coding of the response, which yields misleading outcomes through identification of the textual segments with hidden apprehensions while assigning the same textual segments to several categories utilizing same group patterns as can be observed during response collection into meaningful units. Line-by-line open coding [62] was adopted just to make the responses more vibrant in all possible meaningful directions. Several studies conducted by Refs. [[63], [64], [65]] suggest statistical analysis should be conducted for the analysis of qualitative data, which was followed for this present study.

**Table 7:** Satisfaction-Importance (IS) Analysis for the AV Adoption Intention at selected study area.

Sr. No.	Sub-scales	Importance		Satisfaction		IS Ratings	
		Rating in %	Ranking	Rating in %	Ranking	Rating in %	Ranking
1.	Intention to use (ITU)	70.23	1st	6.85	12th	64.78	1st
**2.**	Perceived usefulness (PU)	31.87	7th	32.87	6th	25.87	8th
**3.**	Perceived ease of use (PEOU)	26.54	8th	28.76	7th	26.89	6th
**4.**	Trust (TRUT)	48.55	2nd	41.72	2nd	27.11	5th
**5.**	Attitudes (ATI)	21.34	10th	27.39	8th	28.95	3rd
**6.**	Perceived Risks (PR)	35.17	6th	44.62	3rd	32.00	2nd
**7.**	Perceived behavioural control (PBC)	39.25	5th	30.04	5th	15.03	10th
**8.**	Subjective Norms (SN)	41.08	3rd	12.32	11th	7.43	11th
**9.**	Compatibility (CMP)	15.99	11th	38.08	4th	27.31	4th
**10.**	Acceptance (ACC)	40.16	4th	78.11	1st	26.77	7th
**11.**	Perceived Benefits (PB)	23.18	9th	20.31	10th	19.40	9th
**12.**	Perceived Concerns (PC)	10.99	12th	21.33	9th	2.98	12th

**Fig. 14:**
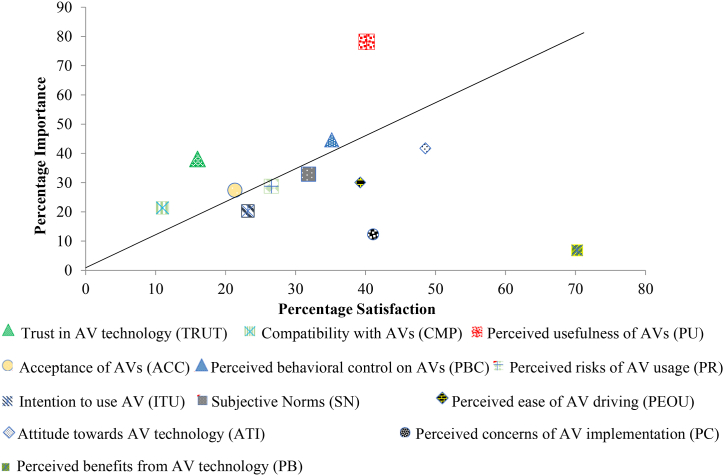
Scatter plot corresponding to satisfaction and importance of AV adoption with reference to 45° slope line.

According to the plot in Fig. 14, sub-scales like perceived benefits (PB), perceived concerns (PC), attitudes (ATI), perceived ease of AV usage (PEOU), social norms (SN), and perceived behavioural control (PBC) all lie below the 45-degree slope reference line (in black through the origin of the 2-D plot), which needs speedy responsiveness, whereas intention to use (ITU) and perceived risk (PR) need immediate consideration while studying AV adoption. Therefore, the aim of our study is to examine all sub-scales that are near a 45-degree slope line, with the aim of validating the perceptions of two-wheeler riders and assessing the importance and satisfaction of AV adoption parameters at a comparable level. Intention to use (ITU), followed by trust (TRUT), has the highest importance rating and the lowest satisfaction rating. This means that the highest number of two-wheeler riders perceive intention to use and trust to be important factors, while they are more dissatisfied by the same. Among the sub-scales, compared in the IS rating; perceived concern (PC) has been assigned the least priority with the lowest IS rating and rank. Most two-wheeler riders perceive compatibility with AVs as the least important, while 38.08 % of two-wheeler is quite satisfied with the rating scales for compatibility with AVs. The reason for this perception is due to the high percentage of educated people participating in the surveys. Fig. 10 depicts the distribution of percentage responses for rating the importance perceived by two-wheeler riders over various sub-scales like intention to use (ITU), perceived usefulness (PU), perceived ease of use (PEOU), trust in AV technology (TRUST), attitude towards AV technology (ATI), perceived risk (PR), perceived behavioural control (PBC), social norms to be followed (SN), compatibility towards AV (COMP), acceptability towards AV adoption (ACC), perceived benefits of using AV (PB) and two-wheeler perceived concerns towards AV technology (PC). Also, a scatter diagram with a 45-degree reference line concentrate fit has been used to ghettoize the factors using satisfaction and importance ratings. The same is depicted in Fig. 14. All factors above the 45-degree reference line often suggest that two-wheeler riders have greater satisfaction for such parameters or sub-scales than importance [66]. On the contrary, parameters below the 45-degree reference line reflect that such parameters need more attention from a two-wheeler rider perspective and have more importance, but riders depict lesser satisfaction and suggest improvements.

The authors also categorized each of the 1673 survey respondents' perceptions into three typical categories: positive, negative, and mixed feedback (mixed representing a cumulative of both positive and negative ones). The perception category (mixed, positive, or negative) is then assigned to each of the 1673 respondents after evaluation of that respondent's overall responses to the open choice constructs shown in Table 3. Depending upon respondent attributes, Table 8 condenses bicycle users and two-wheeler riders' perceptions towards AV adoption intention. Kolmogorov-Smirnov (KS) tests were conducted to identify dissimilarity among the received perceptions. The attributes that were considered for categorizing the types of two-wheeler rider perceptions towards AV usage are: 1. Two-wheeler riders are familiar with AV technology and consider it for transportation of freight and passengers, or as a feeder service in the absence of para-transits such as auto rickshaws, or during emergency situations like pandemics. 2. The reliability of AV technology and its maintenance among two-wheeler riders, who perceive challenges such as a decrease in jobs, particularly in app-based shuttle services, internet issues during trips, and legal proceedings related to the introduction of AV in India. 3. Car ownership status in an average household, 4. Age of two-wheeler riders; and 5. Average monthly income of the participants (two-wheeler and non-motorized or bicycle users) in rupees (INR). The best test statistic (i.e., the D-value) following confidence intervals (of 95 % and 99 %) from the Kolmogorov-Smirnov (K–S) test corresponding to observed and perceived opinions about AV adoption intention for both motorized and non-motorized two-wheeler riders have also been displayed in Table 8.

**Table 8:** Bicycle and Two-wheeler Rider Perceptions towards Perceived risk of AV adoption.

Type of Rider	Attributes	Response to open ended questions (in %)			
		Mixed	Positive	Negative	Sig.-value
**Non-motorized two wheeler**	**AV Technology Familiarity:** No familiarity (N = 422) Familiar Enough (N = 251)	25 15	244 122	153 114	≤0.002** <0.001
**Motorized Two wheeler**	No familiarity (N = 232) Familiar Enough (N = 441)	30 12	169 302	33 127	0.05* 0.34*
**Non-motorized two wheeler**	**Reliability on AV in terms of safety and challenges:** Rating of 1 or 2 (N = 187) Rating of 3 (N = 99) Rating of 4 or 5 (N = 387)	19 5 18	100 64 257	68 30 112	≤0.001** ≤0.03** 0.08*
**Motorized Two wheeler**	Rating of 1 or 2 (N = 105) Rating of 3 (N = 196) Rating of 4 or 5 (N = 372)	18 13 16	29 80 195	58 103 161	0.17 0.44 ≤0.004**
**Non-motorized two wheeler**	**Car ownership:** Yes (N = 310) No (N = 363)	17 13	118 109	175 241	0.08* 0.06**
**Motorized Two wheeler**	Yes (N = 512) No (N = 161)	28 15	281 84	28 62	≤0.001** 0.05
**Non-motorized two wheeler**	**Age:** 18–25 yrs (N = 78) 26–35 yrs (N = 43) 36–45 yrs (N = 77) 46–60 yrs (N = 23) more than 60 yrs(N = 34)	11 3 21 5 8	43 38 26 15 10	24 1 30 3 16	0.02 0.005 ≤0.001** ≤0.001** ≤0.001**
**Motorized Two wheeler**	18–25 yrs (N = 104) 26–35 yrs (N = 184) 36–45 yrs (N = 101) 46–60 yrs (N = 56) more than 60 yrs (N = 37)	14 12 27 44 10	77 69 100 56 187	14 12 27 44 10	0.05* ≤0.001** 0.06 0.002 0.005
**Non-motorized two wheeler**	**Monthly income:** Low (lesser than Rs. 20,000) (N = 97) Medium (Rs. 20,000 – Rs. 50,000) (N = 75) High (More than Rs. 50,000) (N = 99)	23 10 6	45 32 74	29 33 19	≤0.001** 0.005** 0.06
**Motorized Two wheeler**	Low (lesser than Rs. 20,000) (N = 107) Medium (Rs. 20,000 – Rs. 50,000) (N = 83) High (More than Rs. 50,000) (N = 87)	18 15 10	68 47 54	21 21 23	≤0.002** 0.02 0.08*

Kolmogorov-Smirnov (KS) test displayed that participant attributes and perceptions had significant correlations for the significance levels: ** = sig.-value ≤0.10; *** = sig.-value ≤0.05.

### 4.3 Discussions on multivariate analysis and structural equation model results

Reliability of TAM/TPB/IDT constructs and latent UTAUT variables were checked using Cronbach's α. Sampling adequacy was tested using the Kaiser-Meyer-Olkin (KMO) statistic yield value of 0.9112 (>0.7) with Cronbach's alpha value of 0.875 (for p < 0.001) at 95 % confidence levels (from Bartlett's test of sphericity results depicted in Table 9), which states that the behavioural assessment tool proposed in terms of all the 13 constructs is indeed reliable for assessing two-wheeler riders' risk perceptions. In this study, a principal component analysis was performed using an orthogonal varimax rotation for all 59 constructs using Smart PLS. Every term or construct was adopted on a Likert scale (1–5). A threshold value of 0.4 for factor loading was adopted, and the extraction was performed for a fixed number of factors.

**Table 9:** KMO and Bartlett's Test for validity of factorial analysis.

Kaiser-Meyer-Olkin value		0.9112
**Bartlett's Test of Sphericity**	Approx. chi-square	8832.189
	Degrees of freedom (df)	672
	Significance value (p-value)	0.000

Rotated component matrix along with corresponding factor loadings after explanatory factorial analysis using principal component analysis is shown in Annexure B (of Appendix) depicted in Table B1. Table 10 shows the confirmatory factorial analysis results along with the internal validation and consistency of the eleven parameters identified from the EFA. Estimated values of Cronbach's α for most of the factors obtained are more than 0.7 indicating acceptable levels of reliability among selected factors with enhanced internal consistency within the 1673 responses.

**Table 10:** CFA results showing internal validation and consistency.

TAM/TPB/UTAUT Constructs	AVE	CR	C-α
**Intention to use (ITU)**	0.725	0.934	0.928
**Perceived usefulness (PU)**	0.732	0.891	0.879
**Perceived Ease of Use (PEOU)**	0.755	0.902	0.879
**Trust on AVs (TRUST)**	0.791	0.950	0.939
**Attitude towards AV Technology (ATI)**	0.841	0.941	0.932
**Perceived Risk towards AV Adoption (PR)**	0.721	0.886	0.873
**Subjective Norms (SN)**	0.727	0.921	0.906
**Compatibility (COMP)**	0.945	0.982	0.834
**Acceptance for AV (ACC)**	0.654	0.848	0.802
**Perceived Benefits (PB)**	0.782	0.956	0.956
**Perceived Concerns (PC)**	0.768	0.962	0.965

Note: AVE: Average Variance Extracted; CR: Composite Reliability; C-α: Cronbach's Alpha.

According to Table 11, reliability tests for the SEM analysis shows that Cronbach's α of latent constructs identified from SEM for total scale is 0.853, and Cronbach's α of every sub-question range between 0.622 and 0.912. Additionally, CITC values range between 0.298 and 0.845. Recent studies [67] have illustrated if Cronbach's α is more than 0.8, higher reliability of SEM model is attributed while values between 0.6 and 0.7 indicate acceptable limits of reliability. Also, the same authors have also stated that corrected item-total correlation (CITC) should be more than 0.4 with minimum limit of 0.3. As, observed from this study, Cronbach's α is more than 0.6 and every CITC values are greater than 0.3. Hence, the data used for SEM analysis is highly reliable for illustrating direct and indirect relations of constructs.

**Table 11:** SEM reliability statistics for identified latent constructs/attributes.

Latent constructs	Sub-question abbreviation	Corrected Item-total correlation (CITC)	Cronbach's alpha if item deleted	Cronbach's alpha
**Intrinsic Facilitating conditions (FC)**	FC_SQ1_L6	0.734	0.911	0.923
	FC_SQ2_L6	0.654	0.902	
	FC_SQ3_L6	0.641	0.901	
	FC_SQ4_L6	0.665	0.900	
	FC_SQ5_L6	0.574	0.912	
	FC_SQ6_L6	0.707	0.904	
	FC_SQ1_L4	0.717	0.905	0.899
	FC_SQ2_L4	0.515	0.877	
	FC_SQ3_L4	0.765	0.822	
	FC_SQ4_L4	0.845	0.713	
	FC_SQ5_L4	0.602	0.622	
**Effort Expectancy (EE)**	EE_SQ1_L5	0.453	0.687	0.873
	EE_SQ2_L5	0.387	0.754	
	EE_SQ3_L5	0.632	0.699	
	EE_SQ4_L5	0.712	0.802	
	EE_SQ5_L5	0.617	0.809	
	EE_SQ6_L5	0.774	0.911	
	EE_SQ7_L5	0.732	0.854	
**Performance Expectancy (PE)**	PE_SQ1_L4	0.711	0.777	0.858
	PE_SQ2_L4	0.688	0.682	
	PE_SQ3_L4	0.456	0.865	
	PE_SQ4_L4	0.387	0.812	
	PE_SQ5_L4	0.298	0.867	

Intrinsic facilitating conditions (FC) are inhibited by location, organizational and environmental parameters. Environmental parameters consist of attributes related to legal (i.e., public policies towards AV adoption support), economic (i.e. per capita GDP), ecologic (i.e. increased levels of CO or nitrous oxide emissions in districts), meteorological (i.e. day, time, week), social (like influential urbanization), and political (i.e. support towards AV deployment). Locational parameters as per [68] include presence of efficient physical infrastructure (i.e. parking space or available charging docks for AVs) while organizational parameters may include rules and regulations of different public transport operators for shared autonomous vehicles. Effort expectancy (EE) the degree of ease associated with technology adaptation [69]. Performance expectancy (PE) denotes degree to which specific technology can provide benefits to its captive users in performing several functions/activities [70]. Discriminant validity within the structural equation models was assessed after utilizing the well-known Fornell-Larcker criterion [55]. According to this criterion, discriminant validity among TPB/TPB constructs and UTAUT/IDT latent attributes are maintained only when the square root of AVE corresponding to each construct exceeds its correlation with all of the other constructs or latent attributes. Table 12 shows results of the discriminant validity using the former criterion. During analysis, the square roots of AVE were arranged diagonally with the corresponding correlations beneath the diagonal. Square root of AVE for “TW riders' age” as obtained is 0.81 which is higher than corresponding to ITU (0.12), PE (0.11), TW riders’ gender (0.22) and so on. Similarly, square root of AVE for “Gender” as obtained is 0.83 which is higher than trust (0.17), age (0.22), perceived concerns (0.18), ITU (0.15), perceived risks (0.18) and so on. If noticed properly, all diagonal values of square roots over AVE corresponding to each TAM/TPB construct are unswervingly higher than other TAM/TPB/IDT constructs across the table.

**Table 12:** Assessment of discriminant validity using Fornell-Larcker criteria.

Construct	**Age**	**ITU**	**PU**	**PEOU**	**TRUT**	**ATI**	**PR**	**SN**	**CMP**	**ACC**	**PB**	**PC**	**PE**	**EE**	**FC**	**Gender**
**Age**	**0.81**	0.12	0.21	0.11	0.15	0.22	0.16	0.15	0.11	0.12	0.15	0.12	0.11	0.16	0.24	0.22
**ITU**	0.12	**0.85**	0.23	0.29	0.18	0.10	0.24	0.31	0.22	0.17	0.19	0.22	0.16	0.17	0.22	0.15
**PU**	0.21	0.23	**0.87**	0.33	0.27	0.25	0.17	0.16	0.16	0.15	0.14	0.22	0.24	0.15	0.13	0.11
**PEOU**	0.11	0.29	0.33	**0.88**	0.14	0.12	0.11	0.12	0.26	0.19	0.28	0.14	0.12	0.11	0.14	0.11
**TRUT**	0.15	0.18	0.27	0.14	**0.92**	0.21	0.23	0.21	0.20	0.24	0.21	0.17	0.21	0.23	0.16	0.17
**ATI**	0.22	0.10	0.25	0.12	0.21	**0.86**	0.13	0.21	0.13	0.17	0.12	0.22	0.27	0.31	0.16	0.17
**PR**	0.16	0.24	0.17	0.11	0.23	0.13	**0.84**	0.12	0.14	0.11	0.14	0.12	0.16	0.18	0.21	0.18
**SN**	0.15	0.31	0.16	0.12	0.21	0.21	0.12	**0.92**	0.11	0.13	0.15	0.21	0.22	0.24	0.21	0.11
**CMP**	0.11	0.22	0.16	0.26	0.20	0.13	0.14	0.11	**0.81**	0.11	0.16	0.17	0.22	0.24	0.21	0.11
**ACC**	0.12	0.17	0.15	0.19	0.24	0.17	0.11	0.13	0.11	**0.86**	0.12	0.14	0.14	0.15	0.14	0.11
**PB**	0.15	0.19	0.14	0.28	0.21	0.12	0.14	0.15	0.16	0.12	**0.82**	0.13	0.11	0.12	0.15	0.22
**PC**	0.12	0.22	0.22	0.14	0.17	0.22	0.12	0.21	0.17	0.14	0.13	**0.86**	0.16	0.23	0.25	0.18
**PE**	0.11	0.16	0.24	0.12	0.21	0.27	0.16	0.22	0.22	0.14	0.11	0.16	**0.81**	0.22	0.14	0.12
**EE**	0.16	0.17	0.15	0.11	0.23	0.31	0.18	0.25	0.24	0.15	0.12	0.23	0.22	**0.87**	0.23	0.11
**FC**	0.24	0.22	0.13	0.14	0.16	0.16	0.21	0.14	0.21	0.14	0.15	0.25	0.14	0.23	**0.91**	0.13
**Gender**	0.22	0.15	0.11	0.11	0.17	0.17	0.18	0.16	0.11	0.11	0.22	0.18	0.12	0.11	0.13	**0.83**

Note: ITU: Intention to use; PU: Perceived usefulness; PEOU: Perceived ease of use; TRUT: trust towards AVs; ATI: attitude towards AV technology adoption; PR: Perceived risk towards AV adoption; SN: Subjective norms; CMP: compatibility; ACC: Acceptance for AV technology; PB: Perceived benefits; PC: Perceived concerns; PE: Performance expectancy; EE: Effort expectancy; FC: Intrinsic facilitating conditions.

The SEM measurement model in the PLS-SEM framework requires cross-loading calculations for discriminant validity assessment. Table A2 in Annexure A within “Appendix” section of this paper shows every level cross-loading against their corresponding TAM/TPB/UTAUT/IDT constructs. According to Hafeez et al. (2024), every level or sub-question should load higher than others to prove discriminant validity. According to Table A2, TW riders' demographic features like ‘Age’ and ‘Gender’ show different loadings on constructs with ‘Age’ showing ‘0.80’ and ‘Gender’ relatively lesser ‘0.75’ loading higher than cross-loadings with other constructs. Similarly, ‘education levels’ and ‘employment status’ showing sufficiently high loadings and very less cross-loadings with the 12 TPB/TAM/UTAUT/IDT constructs as well as “Gender”. Heterotrait-monotrait (HTMT) ratio is well-established modern era reliability measure towards discriminant validity in SEM analysis. HTMT examines whether specific or theoretically dissimilar questions/metrics are empirically distinct [71]. According [33,71], proof of discriminant validity sustains when HTMT ratios which can compare correlations between same and different traits is less than 1. Hafeez et al., 2024 also suggests than values lesser than 0.9 are good while values lesser than 0.85 illustrate excellent discriminant validity among constructs. The HTMT ratios for all 12 TAM/TPB/UTAUT/IDT constructs along with moderating variables like “Age”, “Gender”, “Education levels”, “Employment status” and “Cultural Context” have been summarized in Table A3 of Annexure A (in “Appendix” section of this paper). An HTMT ratio between “Age” and “cultural context” is ‘0.23’ while the same between “Age” and “ITU” is ‘0.18’. Similarly, HTMT ratios between ‘perceived behavioural control (PBC)’ and ‘perceived ease of use (PEOU)’ is ‘0.30’ while the same between ‘perceived usefulness’ and ‘performance expectancy’ is ‘0.21’. All these ratios are substantially lesser than ‘0.85’ meaning sufficient differentiation between the indicators. Also, HTMT ratio of ‘0.18’ between imperative constructs like ‘facilitating conditions (FC)’ and ‘perceived benefits (PB)’ portray important findings through this research. Twelve constructs are considered, which have a cumulative acceptability of 75.38 % of the total unexplained variance. “Perceived benefits” (defined by 9 levels), “intention to use” (defined by 8 levels), “trust” (defined by 6 levels), “perceived risk” (defined by 5 levels), compatibility” (defined by 3 levels), “social norm” (defined by 4 levels), “perceived usefulness” (defined by 4 levels), “attitude” (defined by 3 levels), “acceptance” (defined by 3 levels), and “perceived ease of use” (defined by 3 levels) explained “13.48 %”, “12.49 %”, “10.12 %”, “7.744 %”, “5.903 %”, “5.35 %”, “5.244 %”, “4.946 %”, “4.872 %”, “2.807 %” and “2.15 %” of unexplained variance as ascertained from EFA. To validate the EFA results, confirmatory factorial analysis (CFA) was performed in Smart PLS 4.0.2. For effective removal of similarity within the content and construct wording, co-variance matrices were added to some of the error measures using modification index values, which improved model fitness. The goodness of fit in CFA is measured in terms of CMIN/DF, Akaike information criteria (AIC), Bayesian information criteria (BIC), comparative fit index (CFI), standardized root mean square (SRMR), and root mean square error of approximation (RMSEA) and Tucker-Lewis Index (TLI). The goodness of fit criteria for SEM validity and reliability is (i.e., CMIN/DF as 3, CFI >0.85, SRMR <0.07, RMSEA <0.06, TLI >0.8). The collected data is reliable as all the values of Cronbach's alpha are greater than 0.8 (acceptable value being greater than 0.7).

Table 13 shows the goodness and badness of fit measures for the proposed mediated and moderated SEM models in this study. According to Table 13, it is evident that all the values of the average variance extracted are greater than 0.7 (acceptable value being >0.5), and the value of the square root of the average variance extracted for each factor is greater than any of its correlations with other factors in the model. Hence, the proposed CFA analysis demonstrated good convergence and discriminant validity. The proposed CFA model yielded a CMIN/DF of 0.858, a CFI value of 0.98, an SRMR value of 0.01, and an RMSEA value of 0.02. It can conclude that the model is reliable, shows satisfactory validity, and possess good model fit indices. Therefore, such a measurement model can be utilized for analysis in the binary and ordinal logistic model. All possible hypotheses have been considered during model development for assessing two-wheeler riders’ adoption intentions and perceived risk towards AV usage. Perceived concerns, perceived benefits, compatibility, social norms, perceived risk, acceptance, and trust all have an indirect effect on the intention to use. Whereas perceived ease of use has both direct and indirect effects on intention to use, which implies that attitude has partial mediation effects of PEOU on ITU. Perceived usefulness has both direct and indirect effects on the intention to use, indicating that both trust and attitude have partial mediation effects of PU on ITU.

**Table 13:** SEM models goodness and badness-of-fit measures.

Relations	Model type	CMIN	DF	CMIN/DF	AIC	BIC	CFI	TLI	RMSEA	SRMR
**Two-wheeler riders' perceived risks (PR) of using AVs**	Mediated	578.68	674	0.858	267.56	523.65	0.98	0.83	0.05	0.04
	Moderated	1113.24	674	1.651	477.64	519.55	0.94	0.81	0.03	0.02
**Two-wheeled riders' behavioural intention to use (ITU) AVs**	Mediated	632.78	674	0.938	489.75	625.45	0.87	0.88	0.02	0.05
	Moderated	1256.76	674	1.864	672.44	727.88	0.92	0.82	0.01	0.01

**Fig. 15:**
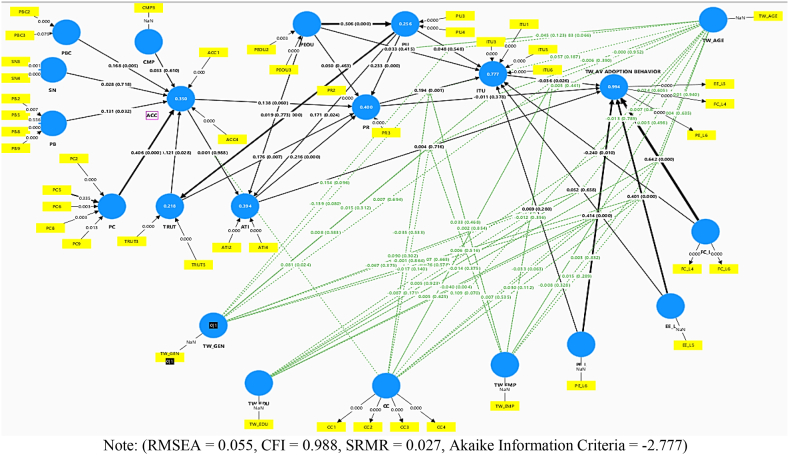
Structural model showcasing TAM-TPB-UTAUT-IDT construct interrelations (Solid arrows represent direct associations; dotted arrows indicate control changes; measured items are in yellow; the TPB/TAM, UTAUT/IDT constructs are in blue; dark ovals indicate high factor loadings and effect size is illustrated with arrow widths) Note: (RMSEA = 0.055, CFI = 0.988, SRMR = 0.027, Akaike Information Criteria = −2.777). (For interpretation of the references to colour in this figure legend, the reader is referred to the Web version of this article.)

SEM path models for two-wheeler riders’ perceived risks and behavioural intention is shown in Fig. 15. According to original sample, sample mean, T-statistics and significance values (P-values) depicted in Table 14, trust (TRUT), intention to use (ITU), attitude towards AV adoption (ATI), and perceived risks (PR) are mostly dominant in influencing AV adoption behavior in India. Although most of the inner model hypothesis (as shown in second column of Table 14) are supported by high T-statistics (greater than 2) and low significance values (P values < 0.05), the relevant correlations and path co-efficient of the outer model with mediation effects can be understood through Fig. 14 and Table 15 more precisely.

**Table 14:** Goodness o fit indices for the TAM/TPB/UTAUT/IDT constructs for SEM model.

Construct	Average variance explained	R-squared value
**Intention to use (ITU)**	0.844	0.509
**Perceived Risks (PR)**	0.802	0.616
**Perceived usefulness (PU)**	0.719	0.615
**Perceived Ease of Use (PEOU)**	0.802	0.609
**Perceived Benefits (PB)**	0.705	0.743
**Perceived Concerns (PC)**	0.766	0.777
**Perceived Behavioural Control (PBC)**	0.700	0.701
**Subjective Norms/Beliefs (SN)**	0.734	0.918
**Acceptance of AV technology (ACC)**	0.722	0.664
**Attitude towards Adoption Behavior (ATI)**	0.705	0.635
**Compatibility with AVs (CMP)**	0.618	0.870
**Performance Expectancy (PE)**	0.734	0.794
**Effort Expectancy (EE)**	0.703	0.802
**Intrinsic Facilitating Conditions (FC)**	0.718	0.762
**Cultural Context/Dissimilarity**	0.719	0.693

**Table 15:** Hypotheses testing original sample and T-statistics from SEM analysis.

Sr. No.	Hypotheses	Original sample (O)	Sample mean (M)	Standard deviation (STDEV)	T statistics ((|O/STDEV|)	P values
Direct effects						
1.	ATI →PR	0.216	0.215	0.062	3.504	0.000
2.	PB → ACC	0.505	0.507	0.074	6.804	0.000
3.	PBC → ACC	0.747	0.735	0.126	5.912	0.000
4.	PC → ACC	0.062	0.061	0.022	2.792	0.005
5.	PEOU → PU	0.401	0.399	0.030	3.331	0.000
6.	PR → ITU	0.240	0.229	0.093	2.570	0.010
7.	PU → ATI	0.642	0.645	0.027	23.994	0.000
8.	PU → PR	0.036	0.034	0.016	2.221	0.026
9.	PU → TRUT	0.131	0.147	0.061	2.141	0.032
10.	TRUT → ACC	0.168	0.163	0.059	2.832	0.005
11.	TRUT → PR	0.406	0.409	0.071	5.702	0.000
12.	ATI → ITU	0.506	0.508	0.042	12.005	0.000
13.	CC → PR	0.414	0.411	0.019	21.668	0.000
14.	PEOU → ATI	0.194	0.193	0.060	3.222	0.001
15.	PEOU → PR	0.171	0.170	0.076	2.264	0.024
16.	PEOU → TRUT	0.233	0.237	0.065	3.572	0.000
17.	PU → ACC	0.467	0.471	0.044	10.684	0.000
18.	PU → ITU	0.121	0.114	0.055	2.199	0.028
19.	PE_L → PR	0.176	0.175	0.065	2.697	0.007
20.	EE_L → ITU	0.075	0.074	0.036	2.089	0.037
21.	FC_L → ITU	0.141	0.136	0.062	2.286	0.022
22.	CC → ATI	0.081	0.081	0.036	2.251	0.024
23.	CC → ITU	0.040	0.041	0.014	2.899	0.004
24.	TW_AGE → ITU	0.412	0.403	0.019	21.769	0.000
25.	TW_EMP → ITU	0.194	0.193	0.060	23.222	0.001
Indirect effects						
1.	PU →TRUT → PR	0.082	0.082	0.031	2.627	0.009
2.	PEOU → PU →PR → ITU	0.109	0.110	0.038	2.895	0.004
3.	PEOU →PU →ATI	0.023	0.023	0.010	2.272	0.023
4.	PEOU →PU →PR	0.021	0.021	0.010	2.154	0.031
5.	PEOU →PU →TRUT	0.087	0.086	0.038	2.262	0.024
6.	ATI →PR →ITU	0.118	0.120	0.035	3.364	0.001
7.	PEOU →PU → TRUT → PR	0.236	0.240	0.032	7.380	0.000
8.	PU → TRUT →ACC	0.042	0.041	0.018	2.378	0.017
9.	PU → PR → ITU	0.045	0.046	0.020	2.301	0.021
10.	CC x ACC → ATI	0.081	0.081	0.036	2.251	0.024
11.	TW_EDU x EE_L → PR	0.040	0.041	0.014	2.899	0.004
12.	SN →ATI → PR	0.027	0.025	0.012	2.170	0.030
13.	CC →ATI → PR →ITU	0.042	0.042	0.017	2.516	0.012
14.	CC → ITU → TW_AV ADOPTION BEHAVIOR	0.056	0.054	0.026	2.153	0.031

Note:ITU = intention to use; PR = perceived risks; ATI = attitudes towards behavior; PU = perceived usefulness; PEOU = perceived ease of use; PB = perceived benefits; PC = perceived concerns; ACC = acceptance; TRUT = trust in AV technology; SN = subjective norms or beliefs; CC = cultural context for Indian districts; PE_L = latent indicator for performance expectancy; EE_L = latent indicator for effort expectancy; FC_L = latent indicator for facilitating conditions in India; TW_AGE = two wheeler riders' age in years; TW_EDU = two-wheeler rider's education levels; TW_EMP = two wheeler riders' employment status measured through monthly income in INR.

Fig. 15 shows correlation and significance results with a summary of the 20 hypotheses around the indicators influencing autonomous vehicle adoption in India. Most of the hypotheses passed correlation analysis showing complex interaction of factors affecting Indian four wheeled autonomous vehicle adoption. Acceptance towards AV technology adoption on the contrary is more correctly predicted by perceived benefits (β = 0.532, for p < 0.001) as compared to perceived risk towards AV usage (β = −0.137, p < 0.001), perceived concerns (β = −0.145, p < 0.001), and compatibility of AV with Indian two-wheeler riders (β = 0.167, p < 0.001), as shown in Table 15. Perceived ease of use influences attitudes the most (β = 0.487, p < 0.001) when compared to acceptance (β = 0.116, p < 0.001) and trust (β = 0.371, p < 0.001). The strongest predictor was perceived ease of use (β = 0.374, p < 0.001) towards AVs intention to use, followed by perceived usefulness (β = 0.256, p < 0.001). All the above findings are consistent enough with studies by Refs. [51,72], while contradicting studies performed by Ref. [73], who argued perceived ease of use does not affect AV intention to use. Table 10 shows standardized mediation/path modeling coefficient estimated using Smart PLS for all possible combination of construct combinations (20 numbers of hypothesis H1 to H20). Mediation effects are generated in SmartPLS software as shown in Table 15. Most of the studies in the past [[72], [74], [75]] have reported self-contradictory results that had unclear remarks about factors that need to be considered for understanding public's opinion regarding AV driving under homogeneous traffic flow conditions in higher-income economies like United States (USA), Germany, and China. At present, there is a deficiency in past literature which suggests factors that probably affect two-wheeler drivers' adoption intentions for AV driving or buying. According to the SEM model path coefficients shown in Table 15, perceived usefulness (PU), perceived ease of AV usage (PEOU), two-wheeler drivers' attitudes towards AV technology (ATI) indeed affect two-wheeler drivers' behavioral intention to use (ITU) AVs even under non-lane-based traffic flow conditions in lower middle-income economies like India. According to Zhang et al. [76], PEOU and PU solely affects AV users' trust for countries like China, the USA, Austria, South Korea, Germany, and France. But from our proposed SEM hypotheses, both PEOU and PU are more likely to affect two-wheeler riders' acceptance of AV technology (ACC) which in turn have indirect total effects on perceived risks towards AV adoption for two-wheeler riders. While PU did affect trust (TRUT), PEOU did not have a direct influence on trust (TRUT), as seen in Table 15. The later statement contradicts findings from studies like [74,72]). Both trust in AV technology (TRUT) and acceptance of two-wheeler riders' (ACC) for AVs, along with compatibility (COMP) and subjective norms (SN) had strong correlations in increasing riders' attitudes (ATI). Riders' attitudes (ATI) in turn had direct effects in judging two-wheeler drivers' behavioral intention to use AV. Perceived risks (PR) and perceived concerns (PC) had a negative effect on Indian two-wheeler riders' acceptance in AVs (ACC) as can be observed from Table 10. The R^2^ of theory of planned behavior (TPB) attribute like perceived risks (PR), intention to use (ITU), perceived usefulness (PU), perceived ease of use (PEOU), acceptance (ACC) and trust (TRUT) as obtained after repeated simulations in SmartPLS are 0.845, 0.769, 0.773, 0.762, 0.757, and 0.802 respectively. A multi-group SEM analysis across all the five districts of Telangana were performed which revealed more robust evidence supporting the theoretical framework of our present study. Table 16 shows a comparative analysis of model fit across the five districts in Telangana state (India) displaying comparative fit index (CFI), Tucker-Lewis index (TLI), root mean square error of approximation (RMSEA), chi-square statistics and significance values (p-values) (see Table 17).

**Table 16:** Results from direct and indirect effects as hypothesized from the SP interview outcomes.

Hypotheses	Standardized path or mediation coefficients	Standard Error	T-value	95 % Bias of Corrected CI	Decision
Direct Effects					
H1**: Perceived Usefulness → Trust**	0.371^^	1.756	2.167	[0.128, 0.478]	Not Supported
H2**: Perceived Usefulness → Perceived Risks**	0.278	0.032	3.628	[0.056, 0.187]	Supported
H3**: Perceived Ease of Use → Perceived Usefulness**	0.191	0.035	4.167	[0.032, 0.298]	Supported
H4**: Perceived Ease of Use → Attitude**	0.489	2.488	2.765	[0.288, 0.743]	Not Supported
H5**: Perceived Ease of Use → Perceived Risks**	0.073^^	0.006	4.917	[0.015, 0.265]	Supported
H6**: Perceived Benefits → Acceptance**	0.531	1.425	4.672	[0.333, 0.611]	Not Supported
H7**: Trust → Perceived Risks**	−0.143^^	0.000	5.109	[-0.108, 0.203]	Supported
H8**: Perceived Concerns → Acceptance**	−0.146	0.035	2.307	[-0.066, 0.189]	Supported
H9**: Compatibility → Acceptance**	0.168^^	0.033	4.176	[0.092, 0.177]	Supported
H10**: Subjective Norms → Trust**	0.089	0.002	4.555	[0.053, 0.144]	Supported
H11**: Acceptance → Trust**	0.216	0.036	2.652	[0.156, 0.447]	Supported
H12**: Acceptance → Attitude**	0.509	3.098	2.187	[0.038, 0.607]	Not Supported
H13**: Trust → Attitude**	0.372	2.098	4.265	[0.024, 0.768]	Not Supported
H14**: Attitude → Perceived Risks**	0.557^^	0.051	4.327	[0.189, 0.622]	Supported
H15**: Perceived Risks → Intention to Use**	−0.412^^	0.046	2.109	[-0.205, −0.688]	Supported
H16**: Perceived Ease of Usefulness → Behavioural Intention to Use**	0.217^^	0.033	4.176	[0.087, 0.333]	Supported
H17**: Perceived Behavioural Control → Behavioural Intention to Use**	0.002^^	1.088	12.98	[-0.001, 0.145]	Not supported
Indirect Effects					
AH1**: Performance expectancy → Perceived risks**	−0.672^^	0.040	2.054	[-0.014, −0.098]	Supported
AH2**: Effort expectancy → Perceived risks → Behavioural Intention to Use**	0.356	0.035	3.011	[0.007, 0.351]	Supported
AH3**: Intrinsic Facilitating conditions → Behavioural Intention to Use**	0.688^^	0.041	4.718	[0.004, 0.074]	Supported

Note: ITU: Intention to use; PU: Perceived usefulness; PEOU: Perceived ease of use; TRUST: trust towards AVs; ATI: attitude towards AV technology adoption; PR: Perceived risk towards AV adoption; SN: Social norm; COMP: compatibility; ACC: Acceptance for AV technology; PB: Perceived benefits; PC: Perceived concerns, ^^significance or p-value <0.001 and rest having significance @<0.005; AH: Additional hypothesis owing to Indirect/latent effects in the SEM models.

**Table 17:** Comparative analysis of SEM fit indices across the four districts in India for autonomous vehicle adoption intention.

Districts	TLI	CFI	RMSEA	Chi-square statistic	Significance value
**Hyderabad**	0.933	0.95	0.03	76.57	0.00
**Warangal**	0.925	0.92	0.04	82.45	0.01
**Nizamabad**	0.904	0.90	0.06	95.73	0.06
**Karimnagar**	0.911	0.92	0.02	86.57	0.03
**Khammam**	0.912	0.91	0.05	90.22	0.04

A consistent higher CFI and TLI values across smart cities like Hyderabad and Warangal (with TLI = 0.933, CFI = 0.95), indicate excellent model fit for these cities. Although, variations in RMSEA values particularly in Khammam (RMSEA = 0.005), highlight that unique regional characteristics influence adoption of AVs within India. Such findings in general indicates the study framework’ strength and signifies its adaptability towards different infrastructural and occupational contexts within India. Therefore, the SEM model has high levels of generalizability and relevance towards policy creation couturier to regional dissimilarities.

All the latter mentioned values obtained are greater than the commonly prescribed limit of 0.76 suggested by Ref. [26]. This indicates that the improved SEM model has better predictable power. Hypotheses H1, H4, H6, H12, H13 and H17 (owing to direct model effects) were rejected by the proposed SEM models due to high standard errors (>1.00) and hence, additional hypotheses AH1, AH2 and AH3 (owing to indirect model effects) were tested. According to Table 16, TAM constructs like perceived usefulness (PU), perceived ease of use (PEOU), perceived benefits (PB), and perceived behavioral control (PBC) does not govern Indian two-wheeler user mind-sets towards wiliness to purchase four-wheeled autonomous vehicles. Accordingly, the indirect effects support the additional hypothesis (AH1, AH2 and AH3) which signify indirect relations of two-wheeler riders' acceptance (ACC), compatibility (CMP) and subjective norms (SN) on perceived risks (PR) while trust (TRUT) can be connoted as the only direct influencer according to theory of planned behavior (TPB) and theory of reasoned action (ToRA) for governing Indian two-wheeler riders' risk perceptions in adopting or buying AVs. This means those drivers’ normative beliefs within the society in terms of hedonic motivation (like reputation and safety) increases trust in using AVs under Indian scenario. This finding is quite contradictory to that found by Zhang et al. [26] for Chinese drivers, which supports utilitarian motivation (like cost and uniqueness) over hedonic motivation (like reputation and safety) towards AV adoption. To portray robustness illustrated by the proposed TAM-TPB-UTAUT-IDT SEM model, K-fold cross validation technique was adopted. Table 18 depicts results of the K-fold cross-validation across five districts (k = 5) of Telangana, India showing significant CFI, TLI, RMSEA, chi-square statistic and significance values.

**Table 18:** K-fold cross validation results across the five Indian cities (k = 5).

Fold	TLI	CFI	RMSEA	Chi-square statistic	Significance value
**1**	0.93	0.96	0.045	76.57	0.04
**2**	0.93	0.96	0.037	86.29	0.02
**3**	0.91	0.92	0.081	92.61	0.06
**4**	0.90	0.91	0.041	88.97	0.01
**5**	0.92	0.93	0.054	82.34	0.03

Acceptance of AV technology (ACC) for two-wheeler riders had more didactic effects (H12:0.509 > H11:0.216, from Table 10) on rider attitudes compared to trust in AV technology, which is consonant with the findings of [74,77] but in contradiction with [28]. Rider's age had a significant effect on AV adoption intention across all perception categories (mixed, positive, and negative). Younger (18–25 years) motorized and non-motorized two-wheeler riders compared to their older counterparts (>46 years) had more positive feedback on AV adoption under Indian disordered traffic flow conditions. High-income [>Rs. 50,000 ($600)] group riders have more propensities towards shifting to AV, while medium income [Rs. 20,000 ($240) –Rs. 40,000($480)] groups do not think AV adoption is uneconomic. It is quite interesting to note that even under such non-benevolent traffic operations with lesser internet connectivity, motorized two-wheeler riders who own at least one private car are still willing to shift to AV if provided with a low-cost option for their shopping and work-based trips.

On the other hand, non-motorized two-wheeler riders feel safer while riding AVs compared to their motorized counterparts (higher number of positive perceptions for rating 4/5: 195 on 161 and 257 on 112). According to the results of the path-based mediation model proposed in Fig. 15, perceived ease of use (PEOU) has the most significant marginal effects on two-wheeler riders' perceived risk of AV adoption, which accounted for more than 20 % of the unexplained variance (with a CMIN/DF of 2.835, CFI of 0.96, an SRMR value of 0.033 and RMSEA of 0.044). According to the analyses presented through structural equation components, interest to adopting or buying autonomous vehicles for two-wheeler riders driving conventional vehicles (petrol/diesel or battery operated) can be measured more by understanding one's intention to use (ITU) behavior rather than understanding their perceived risks (mean value of 2.85 for ITU against 2.55 for PR as shown in Table 19). This finding from the proposed models is not at all unorthodox and unsupportive to the “theory of planned behavior” (TPB) or “technology acceptance” (TAM) [76] principles, as trust (TRUT) towards any technology increases only when there is increased use of that technology towards efficient mobility in daily life. And to be very candid, two wheeler riders in India mostly drive in risky and non-priority based traffic flow conditions in India which over the years have made them more self-reliant and inexorable towards forcing and squeezing through smaller available spatial gaps (sometimes even less than 50 m) during gap acceptance or overtaking under meager congested traffic flow conditions [78,79] Therefore, it can be easily understood that due to the intransigent risk taking attitude of two wheeler riders', their perceived risks (PR), which are clear indicators of the number of fatal AV crashes per year, do not force them to take negative decisions on buying or driving AVs under Indian adverse traffic operational scenarios. This finding is inconsistent with similar studies [26,69,72,80,77] conducted to understand AV user perceptions operating under homogeneous lane-discipline following higher-income economies (like Australia, Germany, USA, China, etc.) where trust (TRUT) in AV technology is more influenced by perceived risks and concerns (PR/PC) than behavioral experience or intention to use (ITU) autonomous vehicles. Table 20 shows that attitude (ATI) and subjective norms (SN) emerged as strong predictors undermining effects of perceived benefits (PB), perceived concerns (PC) and perceived behavioral control (PBC). While facilitating conditions (FC) emerged as strong UTAUT predictor compared to effort expectancy under Indian scenario. Unfortunately, trust (TRUT) in AV technology and acceptance (ACC) of AVs under Indian infrastructure overpower perceived usefulness (PU), perceived ease of use (PEOU) and compatibility (CMP).

**Table 19:** Descriptive statistics of two-wheeler riders’ perceived risks (PR) and behavioral intention to use (ITU) autonomous vehicle (AVs) as obtained from the proposed SEM models.

Dependent variable contributing towards two-wheeler riders' “interest in AVs”	Maximum value*	Minimum value*	Mean value*	Median value*	Mode value*
Perceived risks (PR)	4.00	1.00	2.55	2.05	3.28
Intention to use (ITU)	5.00	2.00	2.85	2.33	3.77
Variables considered	7	7	7	7	7
Sample size	674	674	674	674	674

Note: * - values are determined after conversion of SEM model derived path coefficients to weighted-normalized closeness coefficients using TOPSIS (Technique for Order of Preference by Similarity to Ideal Solution) method in MATLAB (version R2021b) and then ranking using Likert scaling of 1.00–5.00 (with 1 being “unlikely” corresponding to highest closeness coefficient and 5 being “most likely” corresponding to lowest closeness coefficient) assuming PR & ITU as alternatives and PEOU, PU, TRUT, ACC and ATI as decision criteria for all 671 respondents.

**Table 20:** Results of Bayesian SEM analysis for understanding two-wheeler riders’ perceived risks in Indian districts.

Constructs	Posterior average	Standard deviation (SD)	95 % credible interval
Technology acceptance model (TAM) factors			
PU	0.40	0.060	[0.32, 0.54]
PEOU	0.45	0.055	[0.36, 0.57]
Theory of planned behavior (TPB) factors			
ATI	0.85	0.032	[0.81, 0.93]
SN	0.80	0.041	[0.70, 0.90]
Unified theory of acceptance and use of technology (UTAUT) factors			
PE	0.70	0.027	[0.60, 0.80]
EE	0.50	0.055	[0.40, 0.60]
FC	0.75	0.039	[0.71, 0.79]
Theory of innovation diffusion (IDT) factors			
ACC	0.65	0.040	[0.55, 0.75]
CMP	0.55	0.055	[0.48, 0.63]
TRUT	0.60	0.025	[0.50, 0.70]
Socio-economic/demographic factors			
Age (moderating effects)	−0.20	0.023	[-0.27, −0.11]
Gender (moderating effects)	0.10	0.030	[0.06, 0.14]
Education levels (moderating effects)	0.15	0.028	[0.09, 0.21]
Employment status (Gross monthly income) (moderating effects)	0.25	0.020	[0.22, 0.32]

### 4.4 Discussions on random forest analysis results

Bearing in mind aim of the present research, certain questions like “Knowing that you have a fuel i.e. petrol-powered ICE-engine two-wheeler, if you are provided a free ride of a fully automated (i.e., Level-5 automation as per SAE) four-wheeler during your daily commute towards work. Which of the two modes of transport will you choose under present Indian infrastructure?” The outcome of this question was binary out of which 1287 of the respondents chose riding four-wheeler autonomous vehicles (denoted as 0 code in RF) over their two-wheelers while the rest 1386 chose two-wheelers over four-wheeler autonomous vehicles (denoted as 1 code in RF). According to the structural equation models (in Fig. 15), perceived risks (PR) is a better TPB indicator than behavioural intention to use (ITU) for understanding two-wheeler riders’ preference towards willingness to adopt/buy four-wheeled autonomous vehicles in the future. Therefore, the entire dataset of 1673 were split into training (which is 80 % of the imaginative – 1539) used for training the random forest (RF) algorithm while rest 20 % - 1134 were preserved for prediction and evaluation performance of the proposed RF algorithm. A uniform distribution of the dependent variable within the nascent dataset is also retained within the training and testing sub-datasets before splitting process in random forest (RF) as suggested in Kuhn (2008).

**Fig. 16:**
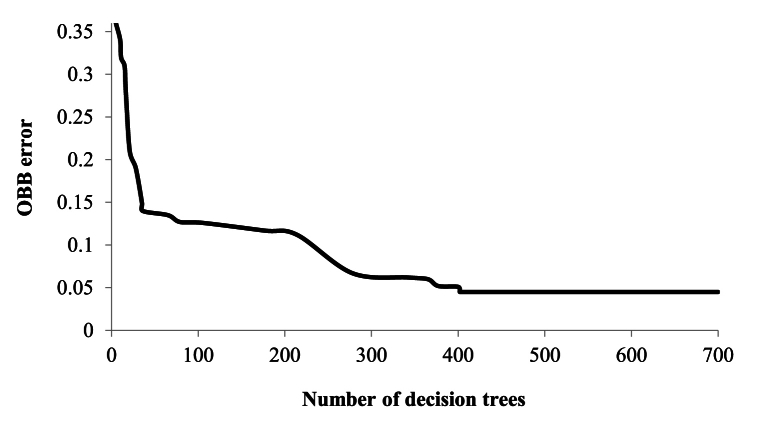
Out-of-bag error (OBB) rate versus number of decision tree plot for the proposed RF models.

The uniform dependent variable classes were maintained among variable class ratios in order to avoid skewness of results due to imbalance in distribution. Initially, a tuning turnover was followed for testing various features, tree numbers and number of variables sampled for each and every node of the trees generated. According to Fig. 16, starting from 402 trees, the out-of-bag (OBB) error is found to be relatively low (with a value of 0.045) while the rate of OBB error becomes stable and lesser sensitive around 402 decision trees. Therefore, 402 decision trees were selected for generating the RF model used for feature selection of the TPB/UTAUT/IDT attributes.

**Fig. 17:**
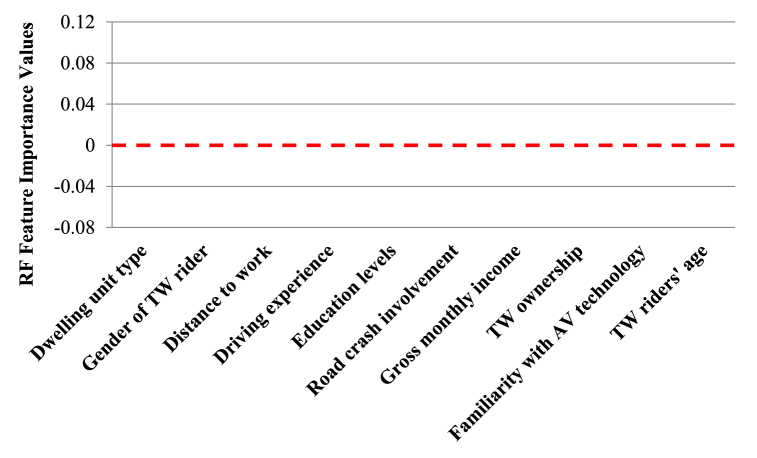
Random Forest feature importance for demographic, socio-economic, and psychological characteristics of SP interview respondents.

**Fig. 18:**
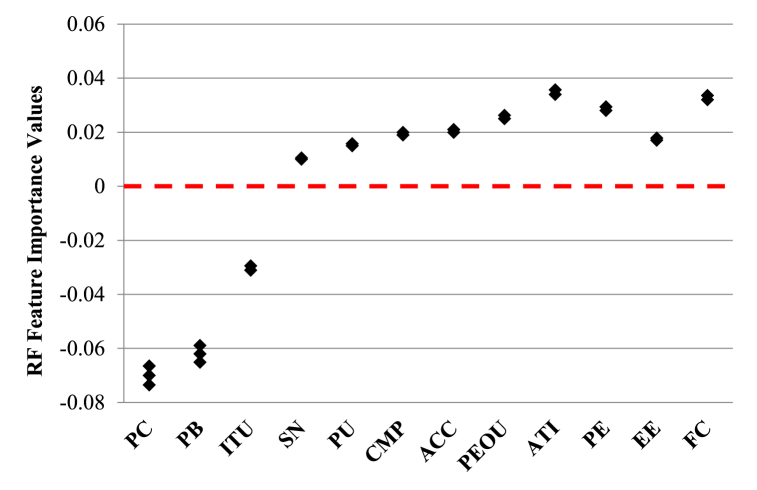
Random Forest Feature Importance for TPB/TAM/TPB/UTAUT factors signifying two-wheeler riders' perceived risks while adopting autonomous vehicles in India.

A final model was proposed based on predictive scoring of including 10 features (corresponding to demographic, travel-behaviour and socio-economic related factors) and 9 features (corresponding to TPB/TAM/TPB/UTAUT factors signifying two-wheeler riders' AV perceived risks). About 500 decision trees with 7 sub-sampled variables were achieved. The proposed algorithm ranked all SP questionnaire variables by feature importance determined through mean decrease in Gini node impurity values as depicted through Fig. 16, Fig. 17, respectively. Quite a lot of observations and discussions can be ascribed based on the RF feature/attribute importance ranks. Firstly, two-wheeler riders' familiarities with autonomous vehicle technology and road crash involvement are the sole psychological factors affecting perceived risks of autonomous vehicle adoption under Indian infrastructure. Among demographic and socio-economic factors, age, number of conventional two-wheelers’ owned (two-wheeler ownership levels) and gross monthly income are the three important features affecting two-wheeler users' behavioural intention to buy autonomous vehicles. On the contrary, distance limitations (i.e., travel time), driving experience in years and gender does not impact the willingness of two-wheeler riders to shift towards AV driving. The latter finding contradicts with similar studies by Refs. [27,47,80,81] which state that males and females respond distinctively in developed countries like USA, Canada, Greece, China, etc. Additionally, education and employment status in Indian scenario are much more important than distance limitations (i.e., travel time) towards influencing AV adoption for two-wheeler users which contrasts findings of Ziakopoulos et al. [27]. According to Fig. 18, Indian two-wheeler users’ rate safety (i.e., involvement in prior road crashes) higher than travel time (distance to work) and infrastructure (dwelling unit type/road conditions) while buying autonomous vehicles (AV) in the future irrespective of travel cost (reflected by gross monthly income).

**Fig. 19:**
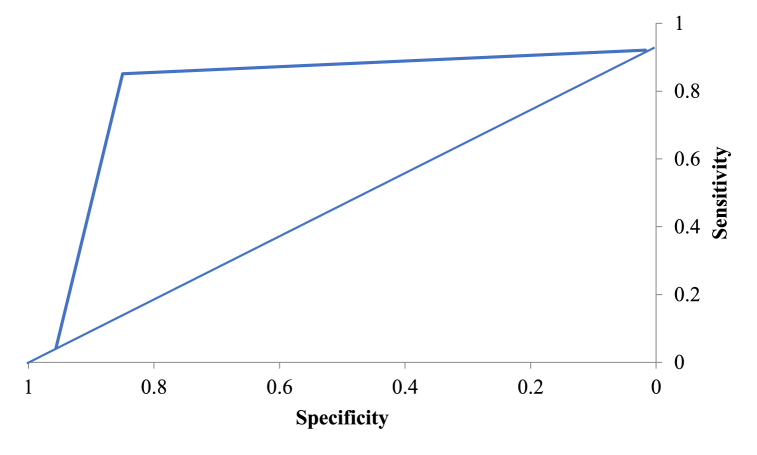
Receiver-operator characteristics (ROC) curve showing Random Forest (RF) prediction accuracy.

According to Fig. 18, TPB/TPB/UTAUT/IDT factors like two-wheeler riders' attitude (ATI), perceived ease of use (PEOU), acceptance to AV technology (ACC), compatibility with self-driving (CMP), perceived usefulness (PU) and normative/social/subjective beliefs (SN) are ranked as 1st, 2nd, 3rd, 4th, 5th and 6th, respectively. While rest of the TPB/TAM/UTAUT factors had comparative lesser contributions while formulation of decision trees in RF. Indicatively, perceived benefits (PB), concerns (PC), intention to use (ITU) and trust in AV technology (TRUT) are not directly related with two-wheeler user autonomous vehicle adoption choices by the survey respondents. Using the testing sub-set data, predictions from the proposed RF model was performed during training. Contrast between binary prediction and actual values as shown in Table 21 were performed. While defining the RF problem, 1 was signified as two-wheeler riders' selecting four wheeled AV over their current conventional one while 0 being encoded as the same two-wheeler riders' sticking to their current mode over four-wheeled AV free ride. A maximum cut-off value of 0.5 was retained while classifying just to ensure uniform predictions. The receiver-operator characteristics curve corresponding to the confusion matrix is depicted in Fig. 19. The area under the ROC curve was found to be 0.851 (matching sum of diagonal elements of the confusion matrix as 85.1 %). This means the RF algorithm did a decent job in predicting the Indian two-wheeler riders’ behavioural intention to buy/drive autonomous vehicles in the future.

**Table 21:** Confusion matrix corresponding to random forest (RF) test subset.

Predicted value of the response	Actual value of response	
	1	0
1	48 (41.20 %)	15 (6.86 %)
0	19 (8.14 %)	56 (43.80 %)

### 4.5 Discussions on TAM, TPB, UTAUT and IDT attribute ranking results based on two-wheeler's risk perceptions

Garrett's ranking technique for verification of the two-wheeler riders' perception across the state of Telangana for all the 15 theories of planned/theory of reasoned action/unified theory of acceptance and use of technology (TAM/TPB/UTAUT/IDT) attributes is conducted. The ranking technique utilized in this study ranks the riders' responses and helps identify the most significant variable or factor affecting user perceptions using rank or percentage [82,77]. Main advantage of this ranking strategy among other frequency distributions is the constructs/factors are arranged or systematized based on their risks from riders' point of view.

The collected riders’ preference/ranks are converted to numeric scores. Now, for conversion, the two-wheeler riders must request ranking of all the factors and additionally all ranking outcomes should be transformed into scores using following Equation (5):

(5) $$\text{PP}=\frac{100\left(r_{\text{pq}}-0.5\right)}{N_{q}}$$

In Equation (5), $r_{\text{pq}}$ is the ranking provided for pth TPB/TAM/UTAUT/IDT factor by qth two-wheeler rider, while $N_{q}$ denotes number of factors ranked by qth two-wheeler rider and PP is the percentage position in the risk perception ranking. Now, according to the SEM results, two-wheeler riders' perceived risks is directly influenced by perceived usefulness (PU), perceived ease of use (PEOU), trust (TRUT) and riders' attitudes (ATT) and indirectly influenced by acceptance (ACC), compatibility (CMP) and riders' subjective norms/normative beliefs (SN). But SEM could not rank the priority of these TAM/TPB/UTAUT factors after mediation analysis. Therefore, Garrett's ranking strategy is utilized for verification of model results regarding the rider responses. To do so, the rider response frequency is determined for assigning rank to each of the 15 factors using the collected data.

Perceived benefits, perceived concerns and perceived behavioural control constructs are utilized for calculating the rider response frequency. The later mentioned three are the sole random values directly indicating risk perceptions of two-wheeler riders in using AVs. Results of the conversion are depicted in Table 22. As observed from Table 22, for perceived usefulness (PU), trust (TRUT), compatibility (CMP) and subjective norms (SN), frequency of 1 and 2 responses are more which means most of the two-wheeler riders feel both compatibility and subjective norms to be more influential than PU and trust under Indian scenario (contradicting findings of [26,47]).

**Table 22:** Frequency of the two-wheeler riders’ responses.

Constructs	Ranking provided by conventional two-wheeler users				
	1	2	3	4	5
Technology acceptance model (TAM) factors					
Perceived usefulness (PU)	15	15	104	200	339
Perceived ease of use (PEOU)	12	15	87	250	309
Theory of planned behaviou**r** (TPB) factors					
Attitude towards behaviou**r** (ATI)	82	168	18	106	299
Subjective norms/beliefs (SN)	58	54	21	219	321
Unified theory of acceptance and use of technology (UTAUT) factors					
Performance expectancy (PE)	70	63	17	125	398
Effort expectancy (EE)	33	11	19	288	322
Intrinsic facilitating conditions (FC)	94	19	8	180	372
Theory of innovation diffusion (IDT) factors					
Acceptance to AV technology (ACC)	58	48	16	188	363
Compatibility with AV technology (CMP)	40	50	65	131	387
Trust on AV technology (TRUT)	55	70	6	142	400

Note: 1 = least influential; 2 = less influential; 3 = can't say enough; 4 = moderately influential; 5 = highly influential.

After this, Equation (5) is used for calculating the percent position of every response as depicted in Table 23. The assigning of ranks for all the TAM/TPB/UTAUT factors is done after multiplying Garrett's value by every TAM/TPB/UTAUT factor frequency of two-wheeler rider response in accordance with the five-point Likert scale (for example, corresponding to TPB factor Trust, 1-point Likert scale = 80* 299 = 23, 920). This rank is assigned depending upon two-wheeler riders' response frequency and corresponding Garrett's value is depicted in Table 23. Then, the risk percentage is determined as total value divided by 1673 (number of responses/data). Garrett's ranking mechanism is governed by high-influence on low-influence phenomenon, according to which a high percentage reveals higher priority of TAM/TPB/UTAUT/IDT factors in influencing riders' risk perceptions in buying autonomous vehicles. Nonetheless, this research captures responses on a 1 to 5 Likert scale (with 5 for highly non-influential and 1 for highly influential) and thus, higher percentage will indicate highly non-influential TAM/TPB/UTAUT/IDT factors in addressing perceived risks (PR). Table 24 shows that Garrett's ranking results are found to be consistent with the Random Forest results and is concluded that two-wheeler riders' attitudes, perceived ease of use and acceptance are the three main TAM/TPB/UTAUT/IDT constructs which drives two-wheeler riders' risk perceptions in adopting or buying AVs more than trust and subjective norms.

**Table 23:** Percent position with corresponding Garrett's value.

Rank	$100\left(r_{\mathrm{pq}}-0.5\right)$	Value Calculated	Garrett Value
1	100*(1−0.5)/7	7.15	80
2	100*(2−0.5)/7	21.43	65
3	100*(3−0.5)/7	35.72	55
4	100*(4−0.5)/7	50	50
5	100*(5−0.5)/7	65	40

**Table 24:** Garrett's ranking value for TPB/UTAUT factors addressing two-wheeler riders' perceived risks towards AV adoption.

Constructs	Ranking provided by conventional two-wheeler user					Total	Percentage (highly influential to least influential)	Rank
	1	2	3	4	5			
Technology acceptance model (TAM) factors								
PU	1200	975	5720	10,000	13,560	31,455	46.738	**10**th
PEOU	960	975	4785	12,500	12,360	31,580	46.924	**9**th
Theory of planned behaviou**r** (TPB) factors								
ATI	6560	10,920	990	5300	11,960	35,730	53.091	**1**st
SN	4640	3510	1155	10,950	12,840	33,095	49.175	**2**nd
Unified theory of acceptance and use of technology (UTAUT) factors								
PE	5600	4095	935	6250	15,920	32,800	48.737	**4**th
EE	2640	715	1045	14,400	12,880	31,680	47.073	**8**th
FC	7520	1235	440	9000	14,880	33,075	49.146	**3**rd
Theory of innovation diffusion (IDT) factors								
ACC	4640	3120	880	9400	14,520	32,560	48.380	**5**th
CMP	3200	3250	3575	6550	15,480	3,2055	47.630	**7**th
TRUT	4400	4550	330	7100	16,000	32,380	48.113	**6**th

Note: PU=Perceived usefulness; PEOU = perceived ease of use; TRUT = trust; ATI = attitude; ACC = acceptance; CMP = compatibility; SN = subjective norms; PE = performance expectancy; EE = effort expectancy; FC = intrinsic facilitating conditions.

## 5 Benefits of the study with policy implications

Adoption and implementation of autonomous vehicles (AVs) in the existing transportation system in mixed traffic conditions is a complex process as there are many factors influencing two-wheeler riders' perceptions. According to recent studies in the past, younger drivers, the majority of whom are technology-savvy males and spent most of their journey in urban environments, have greater interest in this technology and are even willing to pay more for AVs. Some of the two-wheeler riders with automatic driver assistant systems (ADAS) integrated into their vehicles with a past accident history are still willing to shift to AVs. Therefore, we can gain a comprehensive understanding of adoption of this technology by evaluating the acceptance measures of conventional two-wheeler riders, such as their intention or willingness to buy, their trust in AV technology, their ratings on reliability and acceptance, and their perceived attitudes towards AV technology adoption. Previous studies prior to the present one delved around linear explanatory relation between decision constructs using only structural equation models. This study further optimized the linear variations in SEM and validated the same using probabilistic partial proportional odds ratios within the binary/ordinal logit framework in R-studio and ranking the PR and ITU descriptive using TOPSIS method in MATLAB. To the best of authors' knowledge, for the first time, responses of non-license holder two-wheeler drivers along with license-holder responses has been utilized for developing linear structural equation relations between PEOU/PU/TRUT/COMP/ATI and PR/ITU. Additionally, responses have been categorized based on education level, employment status, riding exposure/experience along with order of preferential modes for work-based/shopping-based trips for assessment of both PR and ITU. The introduction of subjective norms (SN) and attitude (ATI) sub-scales for understanding two-wheeler AV usage decisions is quite beneficial as trust (TRUT) had strong latent effects with SN and ATI for two-wheeler riders. The present study also revealed that perceived risks (PR) and trust in AV technology (TRUT) are less crucial compared to riders’ behavioral intention to use (ITU) and attitude towards AVs (ATI), which contradicts the traditional technology acceptance AV adoption intention models (TAM) proposed in the recent past for higher-income economies like the USA, China, Australia, Germany, the UK, Istanbul, and the UAE [37,38,40,[44], [45], [46],^58^].

The stated preference offline interviews (expert opinions and intercept surveys) of two-wheeler riders were conducted during the second wave of the COVID pandemic, where mostly people trusted private modes over public transportation in highly populated countries like India or China. It is often ascertained that utilization of self-reported data from questionnaire may lead towards public response bias [83] which exhibits into individual desirability bias while misunderstanding of questions may lead to recall bias [84]. However, in this study, high respondent magnitude from the present fuel-powered two-wheeler rider population has been collected within the study area to minimize occurrence of such bias. All recall-based questions in our study were transformed to perception and rider belief-oriented questions using generalized discriminant validation-based dimensionality reduction. Therefore, the responses collected can be beneficially utilized for motivating AV adoption in such a calamitous situation. Additionally, the local and regional transport development authorities (like Ministry of Road, Transport and Highways- MORTH and Automotive Research Association of India-ARAI) and emerging private self-driving car developers (such as Swaayatt Robots, AutoNxt Automation, RoshAI, etc.) can gain a deep understanding of two-wheeler riders’ needs, concerns and then formulate optimized policies and novel product development ideas towards two-wheeler driving safety under the Indian traffic flow scenario for the upcoming 10–20 years.

## 6 Limitations of the research

The non-availability of autonomous vehicle (AV) technology in lower-middle income economies and regions like India, Bangladesh, the Maldives, Egypt, and Pakistan makes it difficult to employ the Wizard-of-Oz or ghost driver approach techniques for studying perceived risks towards AV adoption for road users. Therefore, field-based user perception interviews based on video-based techniques are the most suitable options for conducting such studies. On-field or naturalistic driver behavior experiments of AV penetration rates were redundant due to their non-availability under the Indian traffic flow scenario. Although the authors have placed calibrated demonstration videos and figures in the survey questionnaire to let the two-wheeler rider feel comfortable enough with the concept, more responses from a variety of road users (like cycle-rickshaw pullers or people riding tricycles or shared cycles in terms of public bike sharing schemes) and study areas need to be incorporated into the SEM and RF models, considering the bias. Sub-scales and items or constructs formulated for asking open-ended questions need to be increased. As the study was performed in an Indian disordered traffic flow context, the former parameters could not be considered, and hence, in the future (after 10 years), these parameter effects need to be considered in the SEM and RF models. Cost-benefit and infrastructural requirements or parameters towards AV adoption need to be explored in a detailed manner in future studies. In addition to the above limitations, the study did not consider the effects of different levels of automation on perceived risks. Despite such drawbacks in the study, for the first time (under the authors' knowledge), such an extensive study on understanding two-wheeler drivers’ perceived risk towards adoption intention of AVs has been carried out under an Indian disordered traffic flow scenario. The perceived benefits and concerns of two-wheeler riders (bicycles and powered ones) as identified in this study should be communicated in a soothing manner to the public by policymakers, irrespective of ethical dilemma and liability issues. The sub-scales or factors identified from this study will indeed give a push to our policy makers, AV and two-wheeler vehicle manufacturers, in sustainably introducing autonomous vehicles under Indian traffic (which consist of most two-wheeler compositions).

The research generalizes the Indian cultural background, which can be compared with American, German, Chinese, or South Korean cultural backgrounds where there is a sufficient conditional market penetration (over 20 %) of autonomous vehicles and where the two-wheeler rider share is less than 20 % even in urban and sub-urban regions. Such comparative appraisal can yield different response outcomes in terms of interest in AVs and can give a better representation of bicycle or powered two-wheeler riders' cognition and needs towards safer mobility. Besides, future research may also involve the conduct of similar studies considering all 12 sub-scales (PU, PC, PB, TRUT, ACC, ATI, PEOU, PBC, SN, COMP, PR, and ITU) after official penetration of AVs in the Indian market is declared and conventional vehicles (petrol/diesel/battery/electric-operated) start sharing ample space with AVs on the same lane. Also, cross-culture comparison between different lower-middle income regions/economies (World Bank Blogs, 2022–2023) (like India, Bangladesh, the Philippines, Sri Lanka, Nepal, Pakistan, Egypt, the Arab Republic, Bhutan, Uzbekistan, Vietnam, and Cameroon) with varied proportions of two-wheeler shares (ranging between less than 15 % and more than 50 %) may yield more pragmatic sub-scales affecting two-wheeler riders’ AV adoption intention decisions under mixed traffic.

## 7 Conclusions

The study examined attitudes of conventional two-wheeler (TW) users (motorcyclists and bicyclists) and theoretical constructs influencing four-wheeler autonomous vehicle (AV) adoption/purchase intention in suburban regions of India. This research investigates behavioural intension of two-wheeler rider commuters to use AVs using theoretical background of TPB, TPB, IDT and UTAUT. In order to achieve this, twenty-one stated preference field interviews using 40 paraphrased questions was conducted in seven districts of Telangana state of India. A total of 1673 useable responses were retrieved. Explanatory multivariate factorial analysis was carried out to find useful information. Findings depict those twelve important constructs: ITU, PU, PEOU, TRUT, ATI, PBC, SN, CMP, PB, and PC according to TPB, TAM, UTAUT, IDT and TPB affect intention of two-wheeler riders about autonomous vehicles. Structural equation model was proposed using the factors extracted based on the extensions of combined TPB, TAM, UTAUT, IDT and TPB theories in Smart-PLS. Moderation effects based on age, gender, gross monthly income and driving experience were employed on the mediated SEM models. The SEM model was able to demonstrate variable significance at 90 % levels. Various tests and indices under mediation indicate better validity (SRMR, RMSEA), reliability (Cronbach's alpha) and goodness of fit (CFI, TLI) after calibration. Latent variables like performance expectancy (PE), effort expectancy (EE), perceived risks (PR) and intrinsic facilitating conditions (FC) allied towards innovation diffusion theory (IDT) were identified from the SEM model to have sufficient explanatory effects on TW riders' behavioural adoption intention in India. The unsupervised random forest (RF) technique for feature importance analysis was also employed to validate the findings of improved SEM model. The outputs from SEM were used as inputs to the RF algorithm just to compensate the linearity assumption in SEM. Garrett's ranking technique was employed to validate the TAM-TPB-UTAUT-IDT SEM and RF model predictions and identify suitable constructs affecting AV adoption intention in India. Some of the imperative findings from this study are as follows:

1. Descriptive importance-satisfaction (IS) analysis of survey responses indicated that different age groups have varied AV acceptance rates. Over 75 % of younger age group (26–35 years) two-wheeler riders are interested in self-driving while lesser than 20 % older adults (aged more than 60 years) show interest in AVs. These results are in line with [33,85].
2. Survey respondents rated facilitating conditions (in terms of environmental sustainability, regulatory frameworks, infrastructure and safety) higher (87 % relative to 71 %) compared to hedonic motivations (like enjoyment and reputation) for selecting four wheeled AVs over conventional powered two-wheelers. This finding is not in line with studies in south-east Asian countries like China and South Korea [26,74,81].
3. According to our rigorous 21 stated choice experiments, more than 89 % of Indian two-wheeler riders feel fully automated self-driving vehicles will be commercially and politically functional on urban Indian arterials by end of 2040.
4. SEM model showed education levels, employment status, gross monthly income (in INR), age and cultural dissimilarities among the four Indian districts as the key dividends towards AV adoption rates in India. This finding is supportive to previous studies by Refs. [47,86]. Conversely, gender did not have sufficient moderation effects on AV usage behaviour and adoption intentions which contradict findings by Refs. [27,87].
5. According to descriptive statistics based on trust levels and k-cross fold analysis (k = 5) during SEM, Hyderabad (80 %) and Warangal (75 %) showed more confidence in AV adoption compared to Nizamabad (45 %), Karimnagar (48.7 %), and Khammam (42 %). This is due to variation in governmental awareness activities in terms of technology education and infrastructure development. Also, variation in cultural norms among under-developed AMRUT cities like Khammam, Karimnagar, and Nizamabad compared to more liveable smart cities like Warangal and Hyderabad had significant effects on effort expectations of two-wheeler riders. All these findings are in line with cross-sectional and longitudinal AV adoption studies like [6,59].
6. TAM constructs like perceived usefulness (PU), perceived ease of use (PEOU), perceived benefits (PB), and perceived behavioural control (PBC) does not govern Indian two-wheeler user mind-sets towards wiliness to purchase four-wheeled autonomous vehicles. This finding contradicts pivotal American and European AV public acceptance studies [10,25,80,88]. The reason being irrationality of moderation effects due to employment, education levels, gross monthly income levels (price value) in higher economy first world nations like USA, Germany, Canada, and UK unlike India.
7. The theory of planned behaviour (TPB) constructs (attitude towards adoption behaviour and subjective norms/beliefs) ranks higher than UTAUT constructs (performance expectancy, effort expectancy and facilitating conditions) for AV adoption based on Garrett's ranking criteria for Indian two-wheeler riders. This finding contradicts studies underdeveloped higher income economies such as [10,25,80,88]. The reason is the irrationality of moderation effects due to employment levels, education levels, and gross monthly income levels (price value) in higher economy first world nations like USA, Germany, Canada, the UK unlike India.
8. The proposed hybrid extended TAM-TPB-UTAUT-IDT SEM model explained more than 84 % of variances in the study which proves better prediction power compared to previous independent TAM, UTAUT and IDT SEM AV adoption models proposed by Refs. [32,33,44,89,87] all of which could only explain 20 %–70 % of variances.
9. The decision tree based random forest (RF) algorithm could effectively classify the test constructs successfully with a greater degree of precision (over 85 %) compared to former similar studies like [27,55] all of which had lesser than 80 % of accuracy.

10. According to RF feature importance ranking, two-wheeler riders’ age, education levels, gross monthly income (in INR), AV familiarity, history of road cash involvement and two-wheeler ownership levels influence AV adoption patterns in India compared to distance limitations, driving experience and gender. This finding contradicts with similar studies such as [[25], [26], [27],^46^]. Most of the previous studies found gender, driving experience and distance limitations to be most influential compared to age, education and income (price value) for AV adoption.

The study findings listed above can help policy makers, current autonomous vehicle manufacturers and researchers to create a more nuanced approach towards implementing self-driving technology in India. This research also highlighted the importance of cultural context, demography and socioeconomic structure within Indian districts for successful integration of diverse public opinions. Hence, two-wheeler rider age, cost of AV transportation (price value), safety towards AV technology and environmental sustainability should be given prime importance while implementing SAE level-5 autonomous cars on Indian roads.

In the future, three-wheeler and bike taxi user opinions from the same cultural and socioeconomic context along with naturalistic AV driving scenarios on CAV test beds would enhance robustness of the proposed TAM-TPB-UTAUT-IDT SEM and RF models. Additionally, inclusion of additional economic considerations in the SP questions related to purchasing ability of two-wheeler respondents, reliability or price range (on-road price) of Level-5 fully automated vehicles (as per SAE) in Indian market would make the proposed SEM and RF models more robust. All the three parameters is difficult to retrieve at this stage of study and thus, the lost 15 % in RF model prediction is due to such unforeseen factors and partly due to model formulation bias.

## Data availability statement

No secondary data is associated with this research. The questionnaire associated with this research is attached as separate supplementary material with this paper. For primary data sources, information will be shared on request.

## Code availability statement

Smart PLS, R-studio and SPSS-AMOS utilized for creation of the structural equation and random forest models is completely licensed to the author's affiliated institution and therefore not open source.

## Informed consent statement

Signed informed consents for primary data collected in the form of responses from two-wheeler riders have been obtained. The questionnaires were anonymized, and two-wheeler riders were free to opt out of participation in the study whenever they felt uncomfortable.

## Ethical consent statement

The study did not deal with any biomedical and clinical trials on human subjects but still approval from the institute ethical committee with approval number. 45 CFR 46.104/NITW/KADALI/21082 dated 15.01.2022 (before collection of responses from two-wheeler riders) was obtained as one of the co-authors is senior faculty member of the institute itself.

## Funding statement

The work has not been supported by any external or internal governmental or non-governmental funding agencies.

## CRediT authorship contribution statement

**Suprabeet Datta:** Writing – review & editing, Writing – original draft, Validation, Methodology. **Gone Sankeerthana:** Visualization, Software, Investigation, Formal analysis, Data curation. **B. Raghuram Kadali:** Writing – review & editing, Supervision, Resources, Project administration, Funding acquisition, Conceptualization.

## Declaration of competing interest

The authors declare that they have no known competing financial interests or personal relationships that could have appeared to influence the work reported in this paper.
