# Supplementary material for: Do two-wheeler riders propel towards autonomous driving? A path-based probabilistic study in Indian context
Source: Heliyon. 2024 Aug 5;10(15):e35664. doi: 10.1016/j.heliyon.2024.e35664 (PMC11336834; doi:10.1016/j.heliyon.2024.e35664)
Supplement: Multimedia component 1 [file mmc1.docx]

**Part A: Adoption Intention Survey of Independent Automated Driving and Co-operative Urban Road Automated Driving**

Dear Riders/ Drivers,

We will be conducting a survey against knowing your awareness of autonomous vehicle technology, just to understand willingness to buy autonomous vehicles as they are going to enter the car market. This questionnaire is hardly going to take 10 minutes of your valuable time. You are voluntarily free to choose to answer or opt out of this survey. As long as the answer issues true normative beliefs, the survey purpose will be achieved. Thank you for your cooperation in advance.

**Personal Information**

1. **Please mention your gender**

□ Female □ Male

2. **Mention your age:**

3. **Please indicate your marital status:** □ Married □ Unmarried

4. **Please tick your current education level/ status:**

□ Below Elementary level □ Primary school □ High School □ Graduate □ Post-graduate □ University/ College □ Undergraduate □ Masters □ PhD or higher

5. **Are you currently employed?** □ Yes □ No

6. **If, employed, then are you a:** □ Full-time Employee □ Part-time Employee □ Own your business/ start-up

7. **If, employed, please kindly disclose your monthly gross income:**

□ less than Rs. 20,000 □ Rs. 20,000 to Rs. 35,000 □ Rs. 36,000 to Rs. 50,000 □ Above Rs. 50,000

8. **Please select your current occupation from the following?**

□ Student □ Sales representative □ Customer service employee □ Administrative staff □ Clerical (Group-C/ D staff) □ R & D/ technical employee □ Management Employee □ Consultant

□ Teacher/ Faculty □ Professional accountant, lawyer, journalist, medical representative, etc.

□ Others

9. **Do you possess a valid drivers’ license?** □ Yes □ No

10. **How many four-wheelers you own?** □ None □ One □ Two □ More than Three

11. **How many two-wheelers you own?** □ None □ One □ Two □ More than Three

12. **How did you know about autonomous vehicles?**

□ TV broadcast □ Car exhibitions □ Friends or relatives □ I don’t know about AVs □ Newspapers/ magazines □ Through Internet

13. **How long have you been riding your two-wheeler?**

□ less than 2 years □ 3 to 5 years □ 6 to 8 years □ more than 8 years

14. **If employed, how much far is your workplace from your residence?**

□ less than 1 km □ 3 to 5 km □ 5 to 7 km □ more than 10 km

15. **Which mode you prefer while travelling to work?**

□ Private car (4W) □Bicycle □ Public transit □ Motorbike/ Scooty (TW) □ Walking

16. **Which mode you prefer while shopping or visiting your relatives/ friends within your city?**

□ Private car (4W) □Bicycle □ Public transit □ Motorbike/ Scooty (TW) □ Walking

17. **How long have you been driving your private car/ two-wheeler?**

□ less than 5 years □ 5 to 8 years □ 8 to 10 years □ Over 10 years

18. **What do you do mostly while driving your private car/ two-wheeler?**

□ Listen to music □ Eat □ Talk over phone □ Pay attention to road conditions □ Enjoy the off-road habitat □ Frequently look at your phone screen at traffic signals on yellow onsets. □ Others

**Part B: Attitude Survey (Please carefully go through the following description in full)**

**Autonomous Driving based Open-ended Questions**

In India, about 4, 49, 002 road accidents are being reported, among which 151,113 are deaths and 451,361 injuries (MORTH, 2021). Starting in the 1970s, studies (Hilgarter and Granig, 2020; Dingus et al., 2016; Salmon, Regan and Johnston, 2005) indicate human errors like failure; fatigue, impairment, and distraction contribute over ninety percent of accidents worldwide. Recent developments in vehicle technology have deployed autonomous vehicles (AVs) for testing on public roads (Broggi et al. 2015; Shahedi et al. 2023). Autonomous vehicles have the potential to compensate for human errors while driving, which reduces congestion, emissions, and fuel consumption compared to public transit (like BRTS or MRTS) (Salonen and Haavisto, 2019). According to the Mckinsey Center for Future Mobility (2019), the market output value for driverless cars and autonomous driving for the year 2030 within global and regional levels will be expanding to whopping 1.6 trillion dollars, which is almost two times the total revenue generated by leading car manufacturers like Ford, General Motors, Toyota, and Volkswagen for the year 2017. Recent studies (Lu et al., 2017; Fagnant and Kockelman, 2015) indicate an increase in the autonomous vehicle (AV) penetration rate of 75 percent may reduce 95 percent of road accidents. In this respect as a two-wheeler driver we would like to ask your preferences regarding your interest in driving autonomous vehicles in the future under Indian traffic flow conditions. Before filling this section of questionnaire, please go through the following word art showing the SAE j3016 levels of driving automation (Serban et al. 2020) as devised by Society of Automotive Engineers (SAE). DDT refers to Dynamic Driving Task which is a real-time tactical function essential for riding a vehicle and excludes strategic operations like scheduling a trip or planning a specific route.


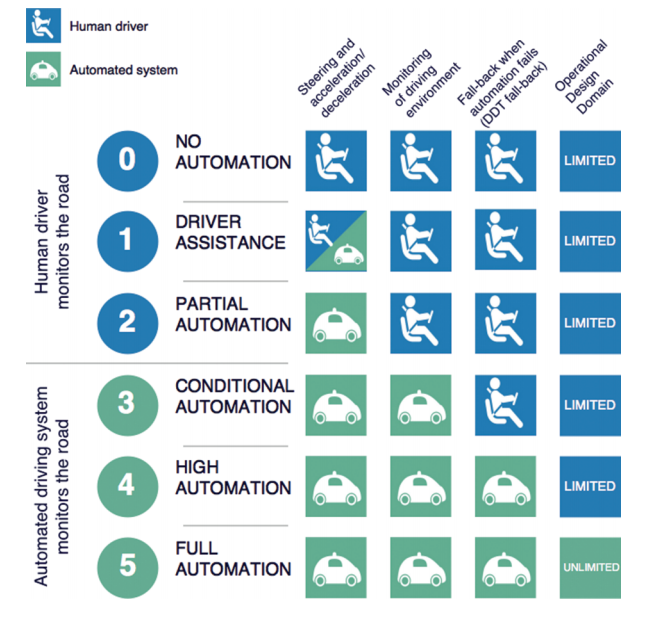


Some of the open-ended questions asked to the respondents:

Please read each one of the questions influencing your decisions to adopt driverless cars adoption compared to your current conventional two-wheeler vehicle and indicate to what extent you agree or disagree with each statement. You can also suggest your valuable remarks or modifications of questions based on your experience with AVs in the past.

You are requested to rate the questions based on the opinion scaling given below:

| **Opinion** | **Scale** |
| --- | --- |
| *Strongly Disagree* | 1 |
| *Disagree* | 2 |
| *Neutral* | 3 |
| *Agree* | 4 |
| *Strongly Agree* | 5 |

| **Sr.no.** | **Questions/Statements** | **Scale** | **Additional Remarks**  **(if any)** |
| --- | --- | --- | --- |
|  | I will be using autonomous vehicles (AV) in place of my current mode of transport. |  |  |
|  | I will be using autonomous vehicles (AV) in the future. |  |  |
|  | I will be buying/ leasing an AV depending upon availability |  |  |
|  | Braking pedals does not affect my AV adoption intention. |  |  |
|  | Maintenance cost affects my AV adoption intention |  |  |
|  | Introduction of advanced technology would help me in adoption of AVs |  |  |
|  | AVs may help increase my driving performance |  |  |
|  | I can relax (listening to music or watch movies) during AV trips compared to conventional two-wheelers |  |  |
|  | AVs will help reduce two-wheeler accidents |  |  |
|  | AVs will help reduce my impairment to drugs, alcohol, etc. |  |  |
|  | It will be easy to ride AVs in case of disability or ill physical health |  |  |
|  | Lack of driving skills will propel me to ride AVs |  |  |
|  | I will buy/ lease AVs for short distance trips (like buying groceries, pharmacies, etc.) |  |  |
|  | I will buy/lease AVs for long distance trips |  |  |
|  | AVs will enhance my driving skills compared to conventional two-wheelers |  |  |
|  | I feel it will be more comfortable to ride AVs compared to controlling conventional two-wheelers |  |  |
|  | AV interaction will increase my mental effort compared to my current conventional vehicle |  |  |
|  | I trust driverless AVs compared to my conventional two-wheeler |  |  |
|  | I feel more safer in my conventional two-wheeler compared to driverless vehicles |  |  |
|  | Modification of government policies may enhance my AV adoption |  |  |
|  | AVs have more reliability compared to conventional two-wheelers |  |  |
|  | AVs are more costly compared to conventional two-wheelers |  |  |
|  | AVs can make more safer decisions compared to any human two-wheeler rider during complex traffic flow situations |  |  |
|  | I will find AVs to be more fun than my conventional two-wheeler |  |  |
|  | It is easy for me to maintain an AV compared to my conventional two-wheeler |  |  |
|  | I want to enhance my knowledge regarding AV operations in absence of conventional two wheelers |  |  |
|  | Indian infrastructure and privacy regulations are supportive to AV technology |  |  |
|  | AVs are more compatible universally than conventional vehicles for all transport systems |  |  |
|  | My driving regime matches AV riding more than my current two-wheeler vehicle |  |  |
|  | AVs may create financial and driver employment losses |  |  |
|  | Sharing same road space with conventional vehicles will make me uncomfortable |  |  |
|  | AVs will definitely reduce vehicular emissions compared to conventional petrol/ diesel operated two-wheelers |  |  |
|  | AVs will reduce my current fuel cost |  |  |
|  | Conventional two-wheelers have lesser maintenance/ ownership costs compared to AVs |  |  |
|  | Driverless two-wheelers may reduce crash potential compared to human rider ones |  |  |
|  | Driverless two-wheelers will have lesser emergency response time compared to human rider ones |  |  |
|  | Driverless two-wheelers will reduce my travel time by 50% |  |  |
|  | A system failure/ service outage/ poor internet connectivity may hamper smooth driverless two-wheeler operations |  |  |
|  | The AV systems attract hacking possibilities and privacy leakage |  |  |
|  | Harsh weather conditions in some parts of India will affect the AV system performance |  |  |
|  | Confusion among connected V2V environment in India during unforeseen situations may hamper my travel itinerary |  |  |
|  | Pedestrians and non-motorized two-wheelers will feel safe in presence of AVs |  |  |
|  | AVs will react faster towards unsafe/ disobeying two-wheeler riders in urban areas |  |  |
|  | I feel safer in my current conventional two-wheeler compared to any driverless options |  |  |
|  | Road markings and signage needs to be improved before AV technology adoption |  |  |
|  | Driverless two wheeler -conventional two wheeler interaction will decrease rear-end collisions at high-speed signalized intersections |  |  |
|  | AVs can detect each and every two-wheeler plying on the entire facility |  |  |
|  | AVs keep two wheeler rider safety as priority compared to human drivers |  |  |
|  | I feel AVs will fail to detect conventional small sized i.e. non-motorized two wheelers on Indian roads |  |  |
|  | I feel Indian political chaos will not let AVs stay for long periods after official market penetration in India |  |  |

This is the end of the questionnaire survey.
